# Functional Properties of Two Distinct PTH1R Mutants Associated With Either Skeletal Defects or Pseudohypoparathyroidism

**DOI:** 10.1002/jbm4.10604

**Published:** 2022-04-14

**Authors:** Ignacio Portales‐Castillo, Thomas Dean, Ashok Khatri, Harald Jüppner, Thomas J Gardella

**Affiliations:** ^1^ Department of Medicine, Division of Nephrology Massachusetts General Hospital, and Harvard Medical School Boston MA USA; ^2^ Endocrine Unit Massachusetts General Hospital, and Harvard Medical School Boston MA USA; ^3^ Pediatric Nephrology Unit Massachusetts General Hospital, and Harvard Medical School Boston MA USA

**Keywords:** HYPERPHOSPHATEMIA, HYPOCALCEMIA, PARATHYROID, PRIMARY FAILURE OF TOOTH ERUPTION, PSEUDOHYPOPARATHYROIDISM, PTH, PTH1R, PTHrP

## Abstract

Consistent with a vital role of parathyroid hormone (PTH) receptor type 1 (PTH1R) in skeletal development, homozygous loss‐of‐function PTH1R mutations in humans results in neonatal lethality (Blomstrand chondrodysplasia), whereas such heterozygous mutations cause a primary failure of tooth eruption (PFE). Despite a key role of PTH1R in calcium and phosphate homeostasis, blood mineral ion levels are not altered in such cases of PFE. Recently, two nonlethal homozygous PTH1R mutations were identified in two unrelated families in which affected members exhibit either dental and skeletal abnormalities (PTH1R‐V204E) or hypocalcemia and hyperphosphatemia (PTH1R‐R186H). Arg186 and Val204 map to the first transmembrane helix of the PTH1R, and thus to a critical region of this class B G protein‐coupled receptor. We used cell‐based assays and PTH and PTH‐related protein (PTHrP) ligand analogs to assess the impact of the R186H and V204E mutations on PTH1R function in vitro. In transiently transfected HEK293 cells, PTH1R‐R186H mediated cyclic adenosine monophosphate (cAMP) responses to PTH(1‐34) and PTHrP(1‐36) that were of comparable potency to those observed on wild‐type PTH1R (PTH1R‐WT) (half maximal effective concentrations [EC50s] = 0.4nM to 1.2nM), whereas the response‐maxima were significantly reduced for the PTH1R‐V204E mutant (maximum effect [Emax] = 81%–77% of PTH1R‐WT, *p ≤* 0.004). Antibody binding to an extracellular hemagglutinin (HA) tag was comparable for PTH1R‐R186H and PTH1R‐WT, but was significantly reduced for PTH1R‐V204E (maximum binding level [Bmax] = 44% ± 11% of PTH1R‐WT, *p* = 0.002). The potency of cAMP signaling induced by a PTH(1‐11) analog was reduced by ninefold and threefold, respectively, for PTH1R‐R186H and PTH1R‐V204E, relative to PTH1R‐WT, and a PTH(1‐15) radioligand analog that bound adequately to PTH1R‐WT exhibited little or no specific binding to either mutant receptor. The data support a general decrease in PTH1R surface expression and/or function as a mechanism for PFE and a selective impairment in PTH ligand affinity as a potential PTH1R‐mutation‐based mechanism for pseudohypoparathyroidism. © 2022 The Authors. *JBMR Plus* published by Wiley Periodicals LLC on behalf of American Society for Bone and Mineral Research.

## Introduction

The human parathyroid hormone (PTH) receptor type 1 (PTH1R) is a class B G protein‐coupled receptor (GPCR) that plays key roles in the development of the skeleton and other tissues, as well as in the homeostatic control of mineral ion physiology. The PTH1R is activated by two distinct endogenous peptide ligands, PTH and PTH‐related protein (PTHrP), which mediate the homeostatic and developmental functions of the receptor, respectively. Homozygous loss‐of‐function mutations in the PTH1R in humans, although rare, lead to the perinatal‐lethal condition of Blomstrand osteochondrodysplasia (BOC),^(^ ^1^, ^2^, ^3^, ^4^, ^5^, ^6^ ^)^ which is mirrored in genetically engineered mice lacking PTH1R.^(^ ^7^ ^)^ More frequently in humans, heterozygous, apparently loss‐of‐function mutations in PTH1R are found in patients with primary failure of tooth eruption (PFE),^(^ ^8^, ^9^, ^10^, ^11^, ^12^ ^)^ supporting a critical role of PTH1R in tooth development.^(^ ^13^, ^14^, ^15^, ^16^ ^)^ Eiken skeletal dysplasia is a rare skeletal condition of delayed ossification that is linked to homozygous PTH1R mutations, and, in two of the three reported families, occurs with PFE.^(^ ^1^, ^17^, ^18^ ^)^ Of interest, patients with Eiken syndrome or PFE are not reported to have defects in maintaining calcium and phosphate homeostasis, nor to have changes in blood levels of PTH, findings which suggest that the developmental pathways regulated by paracrine‐acting PTHrP are more sensitive to the effects of the PTH1R mutations identified in PFE and Eiken syndrome than are the homeostatic pathways that are regulated by endocrine‐acting PTH.

Recently, a novel homozygous PTH1R mutation was identified in a family in which the three affected individuals presented with markedly altered abnormal blood levels of calcium and phosphate, but no reported defect in skeletal or dental development.^(^ ^19^ ^)^ The index case in this nonconsanguineous family was a female who was first diagnosed with epilepsy at age 22 years and then found at age 49 years to have severe hypocalcemia, hyperphosphatemia, intracranial calcifications, mild elevation in serum PTH, and a blunted urinary cyclic adenosine monophosphate (cAMP) response to exogenously administered PTH. The phenotypes suggest a condition similar to pseudohypoparathyroidism type 1b (PHP1B), which is typically linked to epigenetic mutations in the guanine nucleotide binding protein, alpha stimulating (GNAS) locus, which encodes the stimulatory G protein, Gαs.^(^ ^20^ ^)^ Genetic analysis of the index case, however, revealed the homozygous PTH1R missense mutation that changes arginine‐186 in the protein to a histidine (R186H).^(^ ^19^ ^)^ Two siblings of the index case also carry the same homozygous PTH1R mutation, and both presented with elevated levels of PTH, low serum levels of calcium, intracranial calcifications, and no obvious skeletal or developmental abnormality.

Also recently reported is a family with a different novel homozygous PTH1R missense mutation that changes valine‐204 in the receptor to a glutamate (V204E).^(^ ^21^ ^)^ Each of the four homozygous affected sisters in this consanguineous family exhibited delayed tooth eruption, and two exhibited other minor skeletal alterations, including clinodactyly, but there was no reported abnormality in blood chemistries. The phenotypes of the patients with the V204E mutation are consistent with a defect in PTHrP‐mediated signaling during bone and tooth development, whereas the clinical features of the affected members of the R186H family are distinct and suggestive of a defect in PTH‐mediated control of mineral ion homeostasis.

Both the R186H and V204E mutations map to the first transmembrane helix (TM1) of PTH1R, a class B GPCR. As for most class B GPCRs, residues in TM1 of PTH1R are thought to contribute important interactions that are involved in ligand binding as well as the receptor activation process, as suggested by cell‐based functional assays^(^ ^22^ ^)^ and recent high‐resolution structures obtained for PTH1R in complex with a bound PTH or PTHrP analog.^(^ ^23^, ^24^ ^)^ Arg186 and Val204 lie at the extracellular and intracellular ends of the TM1 helix, respectively, and thus would likely contribute differently to the ensemble of intermolecular and intramolecular interactions that underlie the proper folding and function of PTH1R. Mutations at these sites could potentially thus have distinguishable effects on the binding and signaling responses induced by different peptide ligands, and such differences could underlie the divergent clinical pathologies seen in the above two families with the R186H and V204E homozygous PTH1R mutations. To explore this possibility, and the functional consequences of the R186H and V204E amino acid changes, we characterized the properties of both PTH1R mutants in vitro, comparing them to the wild‐type (WT) PTH1R. We included in some of the studies the PTH1R variant with the caveolin‐1 (P132L) mutation, which was identified as a presumably more severe loss‐of‐function mutation in cases of BOC in which both alleles are affected and there is lethality at birth.^(^ ^5^ ^)^

 

## Materials and Methods

### Peptides

Peptides were synthesized by the Massachusetts General Hospital (MGH) peptide core facility using conventional solid‐phase technology with fluorenylmethoxycarbonyl protecting groups and contained C‐terminal amides. Peptides were purified by reverse‐phase high‐performance liquid chromatography (HPLC) to a peptide purity of ~95% as established by analytical reverse‐phase HPLC, and identity was verified by matrix‐assisted laser desorption/ionization (MALDI)‐mass spectrometry. Specific analogs used included: human(h)PTH(1‐34), hPTHrP(1‐36), hPTH(1‐28), hPTHrP(1‐28), hPTH(1‐84),^(^ ^25^ ^)^ hPTHrP(1‐141),^(^ ^26^ ^)^ long‐acting (LA)‐PTH* (AVAEIQL^*n*^ *L*HQRAKWIQDARRRAFLHKLIAEIHTAEY), modified (M)‐PTH(1‐15) (*B*V*B*EIQL^*n*^ *L*HQ^*h*^ *R*AKWY), and M‐PTH(1‐11) (*Z*V*B*EIQLMHQ^*h*^ *R*), where nonstandard amino acids are indicated as: ^*n*^ *L* (norleucine), ^*h*^ *R* (homoarginine), *Z* (aminocyclopentylcarboxylic acid), *B* (aminoisobutyric acid [Aib])^(^ ^27^ ^)^ and standard amino acids are indicated as standard single‐letter code. Fluorescent microscopy experiments utilized tetramethylrhodamine (TMR)‐^*n*^ *L* ^8,21^‐rat‐PTH(1‐34) (PTH(1‐34)^TMR^), in which TMR is attached to the epsilon amino function of lysine‐13. Radioligands used included ^125^I‐M‐PTH(1‐15) and ^125^I‐LA‐PTH*, which were prepared by chloramine T oxidation in the presence of ^125^I‐Na (PerkinElmer, Waltham, MA, USA; NEZ033H; 2200 Ci/mmol) and purified by reverse‐phase HPLC.

### Receptor expression plasmids

Plasmids encoding the hemagglutinin (HA)‐tagged human PTH1R‐WT (TG‐906) and the PTH1R‐R186H (TG‐907) and PTH1R‐V204E (TG‐928) derivatives were generated by VectorBuilder Inc. (Chicago, IL, USA) and contain the cytomegalovirus (CMV) promoter (human cytomegalovirus immediate early enhancer/promoter) driving expression of the PTH1R cDNA sequence and a 3′ SV40 late polyA sequence for transcription termination. The plasmid encoding the HA‐tagged PTH1R‐P132L mutant (TG‐395) was generated in the pCDNA1 vector as described.^(^ ^28^ ^)^ In each PTH1R construct, the nine‐amino acid HA‐tag sequence, YPYDVPDYA, replaces the segment Y^88^PESEEDKE^96^ located in a nonessential region of the N‐terminal extracellular domain (ECD) of the PTH1R, and does not affect receptor function.^(^ ^28^ ^)^

### Cell culture and DNA transfection

GS‐22a cells are derived from HEK293 cells (American Type Culture Collection [ATCC], Manassas, VA, USA; CRL‐1573) by stably transfection with the luciferase‐based GloSensor (Promega, San Luis Obispo, CA, USA) cAMP reporter plasmid pGloSensor‐22F. The cells were cultured in Dulbecco's modified Eagle medium (DMEM) supplemented with fetal bovine serum (10%) in a humidified incubator containing 5% CO_2_ and set at 37°C, and seeded into 96‐well or six‐well plates for transient DNA transfection and functional assays. Cells were transfected when the monolayers were 85%–95% of confluency using Lipofectamine™ 2000 (Thermo Fisher Scientific, Waltham, MA, USA) and 100 ng of DNA per well for 96‐well plates and 1000 ng of DNA per well for six‐well plates. Assays were performed 48 hours after transfection, and media was changed 2–4 hours prior to assay.

### cAMP signaling assays

PTH1R‐mediated cAMP signaling was assessed via the GloSensor cAMP reporter in GS‐22a cells in white 96‐well plates. Assays were performed at ambient room temperature. For each plate, the media was replaced with CO_2_‐independent media (Thermo Fisher Scientific; Cat. No. 18045088) containing luciferin (0.5mM) and the plate was placed into a PerkinElmer Envision plate reader and cAMP‐dependent luminescence, as counts per second (cps), was measured at 2‐minute intervals for 15 minutes, during which time luciferin uptake occurred and luminescence reached a near steady‐state baseline. The plate was then removed from the plate reader, media (vehicle) or test ligands were added at varying concentrations, and luminescence was again measured at 2‐minute intervals for an additional 30–60 minutes. For each well (ligand‐concentration/receptor variant), the peak luminescence signal, which typically occurred 15–20 minutes after ligand addition, was determined and expressed as a percentage of the maximum peak luminescence signal observed in cells transfected with PTH1R‐WT and treated with the same ligand analog.

### Cell surface receptor expression

Chemiluminescent analysis of cell surface receptor expression was evaluated in transiently transfected GS‐22a cells in white 96‐well plates. At 48 hours after transfection, the cells were rinsed with a buffer of Hanks balanced salt solution (Sigma‐Aldrich, St. Louis, MO, USA; Cat. No. H8264) containing 10mM HEPES, pH 7.4/0.1% bovine serum albumin (BSA) (HB), and then incubated in HB containing a horseradish peroxidase (HRP)‐conjugated anti HA.11 antibody (BioLegend, San Diego, CA, USA; Cat. No. 901520) at a final concentration of 1.0 μg/mL (1/1000 dilution) or 0.20 μg/mL (1/5000 dilution) for 1 hour at 4°C. After rinsing twice with HB, chemiluminescent HRP substrate (SuperSignal Pico; Thermo Fisher Scientific; Cat. No. 37070) was added (25 mL/well) and luminescence was read in a PerkinElmer Envision plate reader at 2‐minute intervals for 12 minutes. The cumulative luminescence signal obtained for each well was corrected for background luminescence obtained in wells transfected with pCDNA3.1 vector, and then normalized to the luminescence obtained in wells transfected with PTH1R‐WT, set at 100%.

Flow cytometry analysis of cell surface receptor expression was evaluated in GS‐22a cells that were transiently transfected in six‐well plates and dispersed into a suspension at 48 hours after transfection. The cells were thus enzymatically detached from the wells using TrypLE (Thermo Fisher Scientific; Cat. No. 12563011), suspended in HB (1.0 mL/well), transferred to an Eppendorf tube, and incubated with an Alexa Fluor‐488‐conjugated anti‐HA.11 antibody (BioLegend; Cat. No. 901509) at a concentration of 1 μg/mL for 1 hour at 4°C. The cells were then pelleted by centrifugation, rinsed twice with HB, resuspended in 300 μL HB, and then analyzed in an Attune NxT Flow Cytometer (Thermo Fisher Scientific). Intact single cells were identified and counted by gating on the side‐scattered light channel A (SSC‐A), and cells with bound Alexa Fluor‐488‐anti‐HA.11 antibody were identified and counted by gating on the fluorescence channel (488 nm excitation, 535 nm emission) after setting baseline fluorescence using nonstained cells. For each transfected cell population, values of the mean fluorescence, as relative counts, of the fluorescent gated cells, and their percentage of the total cell population were derived, and after subtracting the corresponding values obtained in cells transfected with pCDNA3.1, were normalized to the corresponding values observed in cells transfected with PTH1R‐WT, set as 100%.

### Radioligand binding assays

Binding of PTH ligands was assessed by competition methods using confluent monolayers of transiently transfected intact GS22a cells in 96‐well plates and ^125^I‐LA‐PTH* or ^125^I‐M‐PTH(1‐15) as tracer radioligands. Reactions were assembled with the plates on ice; and were composed of HB, 20,000–30,000 counts per minute (cpm) of radioligand (2,200 Ci/mmol), and unlabeled competing peptide (M‐PTH(1‐15) for reactions with ^125^I‐M‐PTH(1‐15) and PTH(1‐34) for reactions with ^125^I‐LA‐PTH*) at a concentration ranging from 0.1nM to 1μM. Wells without competing peptide were used to determine maximum binding levels (B0), and wells with the highest concentration of competing ligand were used to determine nonspecific binding (NSB). The plates were then incubated at 4°C for 2 hours, after which the mixtures were removed, the cells were rinsed twice with ice‐cold HB, lysed with 5 N NaOH, and the lysates were counted for gamma irradiation in a gamma counter. Data obtained from reactions with ^125^I‐LA‐PTH* and PTH(1‐34) were processed by subtracting NSB from each measured value at each receptor to obtain specific binding (SB) and dividing by the maximum SB observed in the absence of competing PTH(1‐34) at either the same receptor or at PTH1R‐WT, and plotting the results against ligand concentration to obtain competition binding curves. Curves were fit to the data using a sigmoidal dose–response equation with nonlinear regression (Prism 8.0 software; GraphPad Software, Inc., La Jolla, CA, USA), which, for curves normalized to the maximum specific binding at PTH1R‐WT, provided the negative log of the half maximal inhibitory concentration (pIC_50_) values for competing PTH(1‐34) and the maximum specific of ^125^I‐LA‐PTH* at each receptor as a percentage of the maximum specific binding at PTH1R‐WT.

### Intracellular calcium signaling assays

Signaling via the Galpha‐q (Gq)‐mediated phospholipase C (PLC)/inositol 1,4,5‐trisphosphate (IP_3_)/intracellular calcium (iCa^2^^+^) second messenger pathway was assessed in transiently transfected GS‐22a cells using the calcium‐sensitive fluorophore Fura2‐AM (Invitrogen, Life Technologies Corporation, Grand Island, NY, USA; Cat. No. f1221).^(^ ^29^ ^)^ At 48 hours after transfection, the confluent cells in a black 96‐well plate were preloaded with Fura2‐AM (5μM) in HB for 45 minutes and then rinsed and incubated in HB for 30 minutes. After a final rinse, and on a single‐well‐at‐a‐time‐basis, 90 μL of HB was added to the well and then fluorescence emission at a wavelength (λ_em_) of 515 nm with sequential excitation at wavelengths (λ_ex_) of 340 nm and 380 nm was measured in a PerkinElmer Envision plate reader at 2‐second intervals for 20 seconds prior to (baseline) and for 140 seconds after addition of PTH(1‐34) or PTHrP(1‐36) at a concentration of 100nM. At each time point, the ratio of the fluorescence signal obtained with excitation at 340 nm to that obtained with excitation at 380 nm was calculated and the ratios were plotted versus time.

### Fluorescence microscopy analysis of PTH analog binding and β‐arrestin recruitment

PTH analog binding to WT and mutant PTH1Rs was assessed by fluorescence microscopy in transiently transfected GS‐22A cells as described.^(^ ^30^, ^31^ ^)^ Briefly, the cells were transfected in six‐well plates, reseeded at 24 hours onto coverslips in six‐well plates, and at 48 hours posttransfection processed for analysis. The plates were placed on ice and the cells on coverslips were rinsed with cold HB. Then 1.0 mL of HB was then added to each well, followed by Alexafluor‐488‐labeled mouse monoclonal anti‐HA immunoglobulin G (IgG) antibody (BioLegend; Cat. No. 901509) at a concentration of 1 μg/mL, and the plate was incubated at 4°C for 1 hour. PTH(1‐34)^TMR^ (30nM) was then added and incubations continued at 4°C for 30 minutes. The plate was then shifted to room temperature and incubations continued for 30 minutes. The mixtures were then removed, the cells were rinsed twice with HB, fixed for 5 minutes in 3.7% paraformaldehyde buffer (Boston BioProducts, Inc., Ashland, MA, USA; Cat. No. BM‐158), rinsed with HB, then mounted on a glass microscope slide in Vectashield media containing 4′,6‐diamidino‐2‐phenylindole (DAPI) (Vector Laboratories, Burlingame, CA, USA; Cat. No. H1500) and imaged at magnification ×400 using a Nikon Eclipse fluorescence microscope equipped with a CCD camera configured with SPOT imaging software (Nikon, Tokyo, Japan). Regions of interest were digitally expanded 5× or 7× in PowerPoint software (Microsoft Corp., Redmond, WA, USA).

Recruitment of β*‐*arrestin was assessed in GBR‐24 cells (HEK293/GloSensor (GS‐22A)‐derived cells stably expressing β*‐*arrestin2^YFP^)^(^ ^30^ ^)^ that were transiently transfected to express PTH1R‐WT or a mutant derivative. The cells on coverslips in six‐well plates were treated with PTH(1‐34)^TMR^ (30nM) in HB buffer for 30 minutes at room temperature and then rinsed, fixed for 5 minutes in 3.7% paraformaldehyde, rinsed, and imaged by fluorescence microscopy, as described for GS‐22A cells above.

### ImageJ analysis

Fluorescent microscopy images were analyzed using ImageJ software, ver. 1.53 (NIH, Bethesda, MD, USA; https://imagej.nih.gov/ij/).^(^ ^32^ ^)^ The color images were converted to 8‐bit and then 16‐bit grayscale, and then threshold was adjusted on a cell‐by‐cell basis to show distinct particles of TMR‐labeled PTH(1‐34) together with either HA.11.Alexa488 antibody‐labeled receptors or β*‐*arrestin2^YFP^ within the cell; the threshold setting values ranged from ~50–100. The freehand tool was used to define the area analyzed for each cell, and the Measure>Analyze Particle tool was used to obtain the number of particles, the total area and the mean grayscale (intensity) of each particle for each cell. For each cell, the number of particles was divided by the total area to obtain the Particles/Total Area.

Colocalization analysis of β‐arrestin2^YFP^ and PTH(1‐34)^TMR^ was performed using the JACoP ImageJ plug‐in, which provided Pearson correlation scores for selected cells.

### Data analysis

Data were processed using Microsoft Excel and GraphPad Prism 8.0 software and analyzed statistically using a Student's *t* test (two‐tailed and unequal variances). Dose–response curves were fit to the data by using a three‐parameter sigmoidal dose–response equation with nonlinear regression, which yielded the reported response parameters of potency (EC_50_), efficacy (Emax), and affinity (IC_50_). Data are reported as means ± standard error of the mean (SEM) of cumulative results from three or more independent experiments.

## Results

### cAMP responses of PTH1R mutants to PTH(1‐34) and PTHrP(1‐36)

**Fig. 1:**
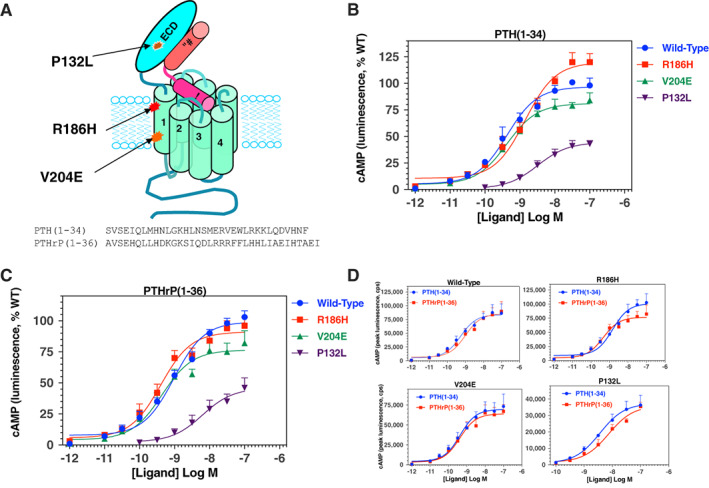
PTH1R mutations and impact on cAMP signaling responses to PTH(1‐34) and PTHrP(1‐36). (*A*) Schematic of the human PTH1R showing the location of the three disease‐causing mutations (P132L, R186H and V204E) and bound PTH(1‐34); amino acid sequences of PTH(1‐34) and PTHrP(1‐36) are shown below. (*B*) cAMP signaling responses to PTH(1‐34) in GS‐22a (HEK293/GloSensor) cells transiently transfected to express PTH1R‐WT, PTH1R‐R186H, or PTH1R‐V204E. Time‐dependent increases in cAMP‐dependent luminescence were measured in a PerkinElmer Envision plate reader following addition of PTH(1‐34), and the peak signal observed on each receptor and each ligand concentration, occurring ~10–20 minutes after ligand addition, was obtained and normalized to the maximum peak luminescence signal obtained with PTH(1‐34) on PTH1R‐WT (100%) and plotted versus ligand concentration. Cells without ligand are represented by the −12 Log M concentration. (*C*) Cells were treated as in *B*, but with varying concentrations of PTHrP(1‐36) and responses were normalized to the maximum response observed for that ligand on PTH1R‐WT. (*D*) The non‐normalized luminescence data, as counts per second (cps), from the experiments of *B* and *C* are replotted to compare the responses observed for PTH(1‐34) versus PTHrP(1‐36) on each receptor. The *y* axes of each graph are adjusted to best display the response at each receptor. Data are means (±SEM) of five experiments. Curves were fit to the data by nonlinear regression analysis; the corresponding potency, maximum and minimum values are reported in Table 1 and Supplementary Table S1.

Because the clinical phenotypes associated with the homozygous PTH1R mutations of R186H (PHP), V204E (PFE) and P132L (BOC) are profoundly different, and the mutations map to distinct sites in the receptor protein (Fig. 1*A*), we sought to determine whether the mutations would result in discernible differences in receptor function in vitro. We first examined the ligand‐dependent cAMP signaling capacities of the mutant receptors using an HEK293‐derived cell line (GS22a) in which the cells stably express a luciferase‐based GloSensor cAMP reporter^(^ ^33^ ^)^ and were transiently transfected with either the WT PTH receptor (PTH1R‐WT) or a disease‐associated mutant PTH1R, PTH1R‐R186H, PTH1R‐V204E, or PTH1R‐P132L, prior to assessing changes in intracellular levels of cAMP, measured as luciferase‐derived luminescence, in response to varying concentrations of PTH(1‐34) or PTHrP(1‐36). We used these two ligands initially as they exhibit high affinity and high signaling potency on the PTH1R‐WT and are thought to replicate at least most of the signaling actions of the endogenous PTH(1‐84) and PTHrP(1‐141) polypeptides that likely function on PTH1R in vivo. Our hypothesis based on the clinical presentation of these two families was that PTH1R‐R186H would have impaired response to PTH(1‐34) and V204E to PTHrP(1‐36).

Cells transfected with PTH1R‐WT or the PTH1R‐R186H or PTH1R‐V204E mutants exhibited dose‐dependent increases in cAMP‐dependent luminescence in response to either ligand that were each robust, and only subtle differences could be detected between the two mutants and PTH1R‐WT (Fig. 1*B*,*C* and Supplemental Table S1). Comparable potencies between the two mutant receptors and PTH1R‐WT were also observed with the full‐length PTH(1‐84) and PTHrP(1‐141) polypeptides (Supplemental Fig. S1 and Supplemental Table S2). In contrast, cells transfected with the PTH1R‐P132L mutant exhibited responses to both ligands that, as compared to the responses on PTHR‐1‐WT, were markedly and significantly (*p* < 0.005) diminished for both potency (negative logarithm of the half‐maximal effective ligand concentration [pEC_50_] ≥ ~8.5 versus 9.36 ± 0.2 for PTH1R‐WT) and efficacy (response maximum or maximum effect [Emax] ~45% that of PTH1R‐WT; Fig. 1*B*,*C*, Table 1). On the PTH1R‐R186H mutant, the maximum response to PTH(1, 2, 3, 4, 5, 6, 7, 8, 9, 10, 11, 12, 13, 14, 15, 16, 17, 18, 19, 20, 21, 22, 23, 24, 25, 26, 27, 28, 29, 30, 31, 32, 33, 34) was moderately higher than that observed on PTH1R‐WT (Emax = 118% ± 4%, *p* = 0.002, Table 1), whereas the potency of the response tended to be slightly (approximately threefold) weaker than that of the ligand on PTH1R‐WT. The reason for the increase in Emax is not clear, because the patient phenotype predicts a deficiency in responsiveness to PTH, which is at least suggested by the trend toward reduced potency. In contrast, PTHrP(1‐36) induced a response on PTH1R‐R186H that was enhanced in potency by approximately twofold, relative to that on PTH1R‐WT (pEC_50_s = 9.40 ± 0.12 versus 9.01 ± 0.98, respectively, *p* = 0.03) with a response maximum comparable to that of PTH1R‐WT (96% ± 2% that of PTH1R‐WT; *p* = 0.08, Fig. 1 *C*, Table 1). On the PTH1R‐V204E mutant, the potencies of the responses induced by either ligand were comparable to, or, for PTHrP(1‐36), enhanced approximately twofold, relative to those induced on PTH1R‐WT, but for each ligand the response maximum was significantly diminished (Emax = 81% ± 4% that of PTH1R‐WT for PTH(1‐34), *p* = 0.003; 77% ± 6% that of PTH1R‐WT for PTHrP(1‐36), *p* = 0.004; Fig. 1*B*,*C*, Table 1).

**Table 1:** cAMP Dose–Response of PTH(1‐34) and PTHrP(1‐36) on WT and Mutant PTH receptors

	PTH(1‐34)				PTHrP(1‐36)					
Parameter	pEC_50_	*p* versus WT	Emax (%)	*p* versus WT	pEC_50_	*p* versus WT	*p* versus PTH	Emax (%)	*p* versus WT	*p* versus PTH
Wild‐type	9.36 ± 0.20 (0.44nM)		100 ± 0		9.01 ± 0.09 (0.98nM)		0.15	100 ± 0		0.69
R186H	8.92 ± 0.13 (1.19nM)	0.11	118 ± 4	0.0024	9.40 ± 0.12 (0.40nM)	0.030	0.028	96 ± 2	0.077	0.001
V204E	9.49 ± 0.13 (0.33nM)	0.61	81 ± 4	0.0029	9.37 ± 0.09 (0.43nM)	0.022	0.48	77 ± 6	0.0041	0.57
P132L	8.46 ± 0.10 (3.45nM)	0.004	44 ± 4	<0.0001	8.21 ± 0.11 (6.15 nM)	0.0004	0.12	45 ± 7	<0.0001	0.95

cAMP‐dependent luminescence responses to PTH(1‐34) and PTHrP(1‐36) were assessed in GS‐22a cells expressing either the WT or a mutant PTH1R. Potency values, as the negative logarithm of the half‐maximal effective ligand concentration (pEC_50_) with the corresponding nanomolar (nM) concentration in parentheses, and the response maximum (Emax), as a percent of the maximum luminescence response observed for each ligand on PTH1R‐WT, were derived from curve fitting dose–response data to a sigmoidal nonlinear regression equation. The corresponding luminescence values for the response maxima and observed minimum are reported in Supplementary Table S1. Data are means (±SEM) of five experiments: *p* values are Student's *t* test comparisons to PTH1R‐WT or to PTH(1‐34).

Comparison of the relative potencies of PTH(1‐34) versus PTHrP(1‐36) on each receptor variant revealed a modest yet significant shift of the two ligands on the PTH1R‐R186H mutant, because PTH(1‐34) was approximately threefold less potent than PTHrP(1‐36) (pEC_50_s = 8.92 ± 0.13 versus 9.40 ± 0.12, respectively, *p* = 0.001, whereas on PTH1R‐WT, PTH1R‐V204E, and PTH1R‐P132L, PTH(1‐34) tended to be slightly (approximately twofold) more potent than PTHrP(1‐36) (Fig. 1 *D*, Table 1). A moderate decrease in response potency to PTH(1‐34) but not PTHrP(1‐36) on the R186H mutant is consistent with the phenotype of pseudohypoparathyroidism without skeletal abnormalities seen in the patients with this mutation. The findings overall support a strong loss‐of‐function impact of the P132L mutation,^(^ ^2^ ^)^ and relatively milder loss‐of‐function effects of the V204E and R186H mutations in the PTH1R, and are consistent with the lethal phenotype associated with the homozygous P132L mutation in BOC^(^ ^2^, ^4^, ^5^ ^)^ and the milder nonlethal skeletal phenotype and/or mineral ion abnormalities encountered in patients with the homozygous V204E or R186H mutations.

### Cell surface expression of WT and PTH1R mutants

**Fig. 2:**
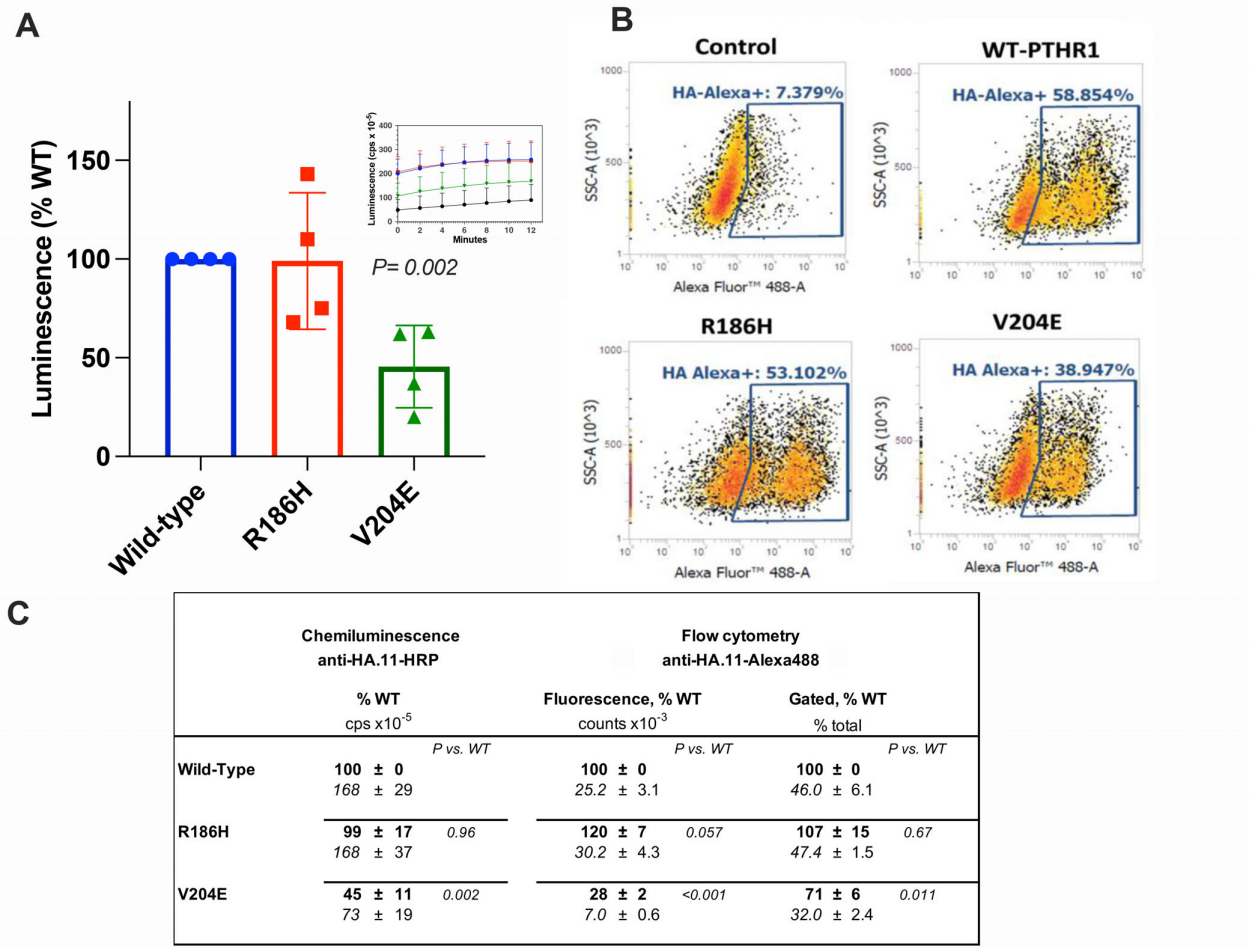
Cell surface expression of WT and mutant PTH receptors. GS22a cells transiently transfected with PTH1R‐WT, PTH1R‐R186H, or PTH1R‐V204E, each with an extracellular HA tag, or pCDNA3.1 (control), were assessed for binding anti‐HA antibody (HA.11) conjugated to either horseradish peroxidase (HRP) for chemiluminescence detection, or to AlexaFluor488 for fluorescence detection by flow cytometry. (*A*) Chemiluminescence of HRP‐anti‐HA.11 antibody bound to confluent cells was measured in black 96‐well plates using an Envision plate reader. After antibody incubation (1 hour at 4°C) and HRP‐substrate addition, luminescence, as counts per second (cps), was measured at 2‐minute intervals for 12 minutes (inset) and the mean cumulative luminescence obtained for each receptor was corrected for the mean luminescence in pcDNA3.1‐transfected cells (black trace in inset) and normalized to the corresponding value obtained for PTHR‐WT (100%). The experiment was done at both a 1/1000 and a 1/5000 dilution of antibody, and similar relative changes in signal for the mutants versus the WT receptor were obtained at each dilution; data displayed are for the 1/1000 dilution. Results for the 1/5000 dilution, as the percentage of signal observed at PTH1R‐WT, were: 97 ± 22 for PTH1R‐R186H and 47 ± 16 for PTH1R‐V204E (*p* = 0.9 and *p* = 0.03, respectively, versus PTH1R‐WT). (*B*) Fluorescence of AlexaFluor488‐conjugated anti‐HA.11 antibody bound to single cells in suspension was assessed by Flow cytometry. For each transfected cell population, the single cell fluorescence is plotted versus the count of individual cells, detected as side‐scattered light (SSC‐A), and the percentage of HA‐Alexa‐fluorescence‐positive cells, contained within the gated area enclosed by the blue lines, was quantified (shown within each graph). Data are from a single experiment representative of three. (*C*) Summary of the expression studies depicted in *A* and *B*; values in bold font are background‐subtracted and normalized to the corresponding value obtained with PTH1R‐WT (100%, font), and those directly below in plain font are the total observed values of HRP‐chemiluminescence (cps × 10^−5^), Alexa488 fluorescence (relative counts × 10^−3^), and percentage of cells gated. The background values observed in cells transfected with pCDNA 3.1, were 71 ± 57 × 10^−5^ cps for HRP‐chemiluminescence, 6.9 ± 1.0 × 10^−3^ counts for Alexa488 fluorescence, and 5.4 ± 2.0% for gated cells. Values of *p* show Student's *t* test comparisons to PTH1R‐WT. Data are means ± SEM of three (AlexaFluor488) or four (HRP) experiments.

The levels of expression of the PTH1R‐WT and the PTH1R‐V204E and PTH1R‐R186H mutants on the surface of transiently transfected HEK293 (GS22a) cells were assessed using an antibody (HA.11) directed against an HA epitope tag that was incorporated into the extracellular domain of each receptor. Expression of the PTH1R‐P132L mutant was not examined here, but previous studies using a green fluorescent protein (GFP)‐tagged variant in COS‐7 cells suggested levels comparable to PTH1R‐WT.^(^ ^6^ ^)^ Figure 2*A*,*C* show the results of a chemiluminescence‐based analysis of intact adherent cells in a 96‐well plate using the HA.11 antibody conjugated to HRP. The PTH1R‐R186H mutant was expressed on the cell surface at the same level as PTH1R‐WT (*p* = 0.96) whereas PTH1R‐V204E was expressed at ~45% the level of PTH1R‐WT (*p* = 0.002). Comparable results were obtained using fluorescence‐activated flow cytometry and staining with an HA.11 antibody conjugated to AlexaFluor488 to analyze expression of the PTH1R variants on the surface of cells in suspension (Fig. 2*B*,*C*). This analysis provided both the fraction of cells in the total population that were gated as AlexaFluor488‐fluorescence‐positive, as well as the mean fluorescence of the individual cells in that gated population (Fig. 2*C* presents the two parameters normalized to PTH1R‐WT). Both of these parameters were slightly though not significantly increased for PTH1R‐R186H (by 20% and 7% relative to PTH1R‐WT for mean cell fluorescence and fraction gated, respectively). In contrast, both mean cell fluorescence and the fraction of cells gated as fluorescent‐positive were significantly reduced for PTH1R‐V204E (to 28% ± 2% that of PTH1R‐WT, *p* < 0.001 and to 71% ± 6% that of PTH1R‐WT, *p* = 0.01, respectively).

**Fig. 3:**
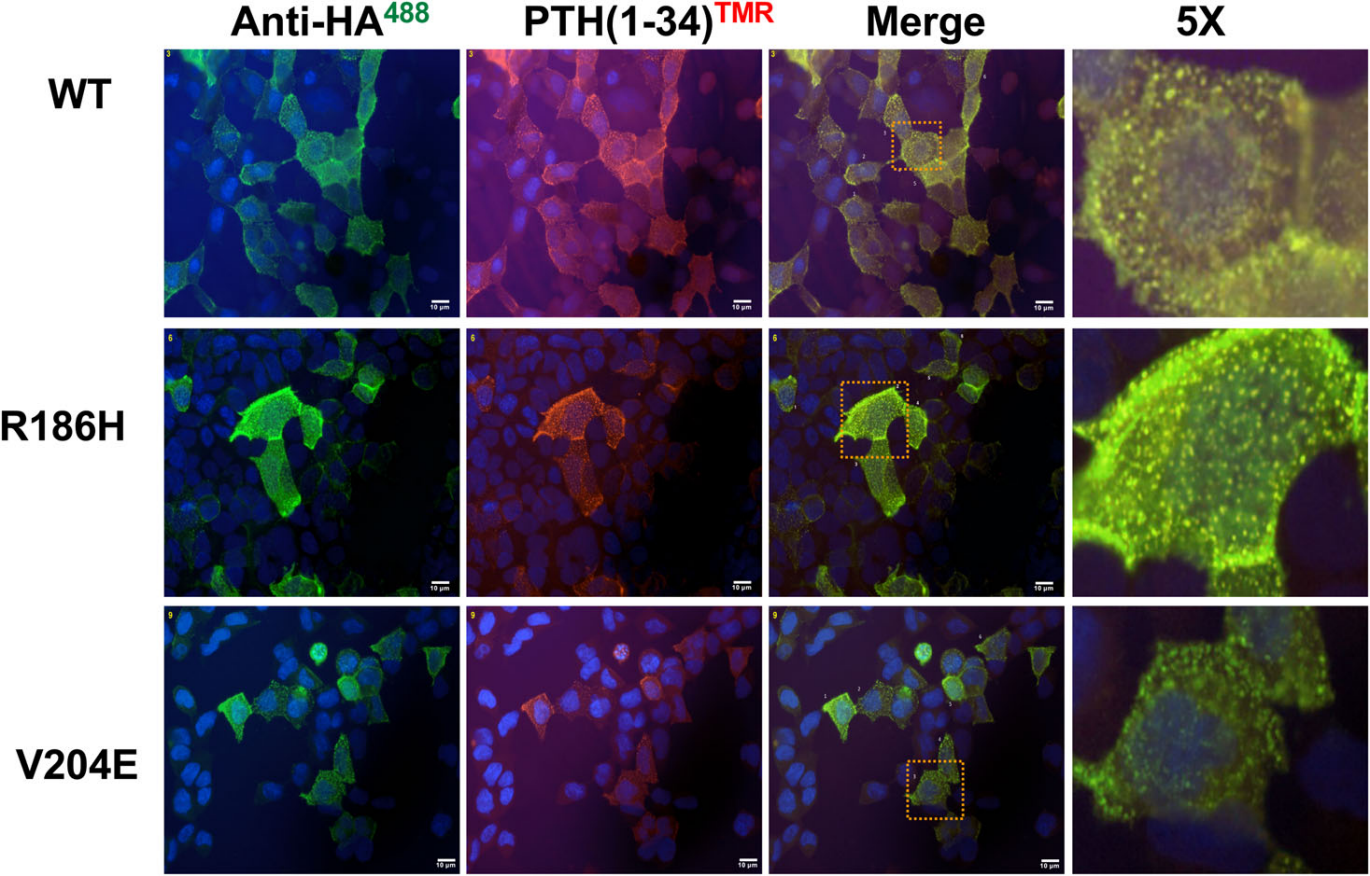
Fluorescent microscopy of receptor cell surface expression and PTH(1‐34)^TMR^ binding. GS‐22a cells transiently transfected to express PTH1R‐WT, PTH1R‐R186H, or PTH1R‐V204E were treated on coverslips with AlexaFluor488‐conjugated anti‐HA.11 antibody for 60 minutes at 4°C, then with PTH(1‐34)^TMR^ (30nM) for 30 minutes at 4°C, followed by a shift to room‐temperature for a final 30 minutes. The cells were then rinsed, fixed, stained with DAPI, and imaged using a fluorescence microscope (magnification = ×400). Transfected cells stain positively for both HA.11‐Alexa488 (green) and PTH(1‐34)^TMR^ (red), which appear to co‐localize along the cell perimeter and to internalized vesicles for each receptor variant, with the signals qualitatively weaker for the PTH1R‐V204E mutant. The rightmost column shows 5× enlarged views of the boxed regions in the merged images. ImageJ analyses performed on selected cells in the fields shown are presented in Supplemental Table S3.

For the purpose of evaluating cell surface expression and ligand internalization, we assessed qualitative immunofluorescence microscopy (Fig. 3). Transfected GS22a cells were incubated on ice for 30 minutes with green‐fluorescent AlexaFluor488‐anti‐HA.11 antibody followed by 60 minutes with a red‐fluorescent TMR‐modified PTH(1‐34) analog, prior to shifting the cells to room temperature for a final 30 minutes to enable PTH ligand‐induced receptor internalization, and then fixing and imaging by fluorescence microscopy. Cells transfected with the three different PTH1R receptors exhibited strong AlexaFluor488‐derived fluorescence that was largely co‐localized with PTH(1‐34)^TMR^‐derived fluorescence at both the cell perimeter and in clusters that were likely to be at least partially internalized within the cells. Consistent with the quantitative antibody binding data of Fig. 2, ImageJ software analysis of individual cells in the images of Fig. 3 revealed a lower number of particles (vesicles) and a lower particle signal intensity for the PTH1R‐V204E mutant than for PTH1R‐WT (Supplemental Table S3). A lower relative expression of PTH1R‐V204E was also observed in nonstimulated HEK293/βarrestin2^YFP^ cells (GBR‐24 cells, see following section) using an HA.11 antibody conjugated to AlexaFluor594 (Supplemental Fig. S1).

### β‐arrestin recruitment and PLC/IP3/intracellular calcium signaling by WT and PTH1R mutants

**Fig. 4:**
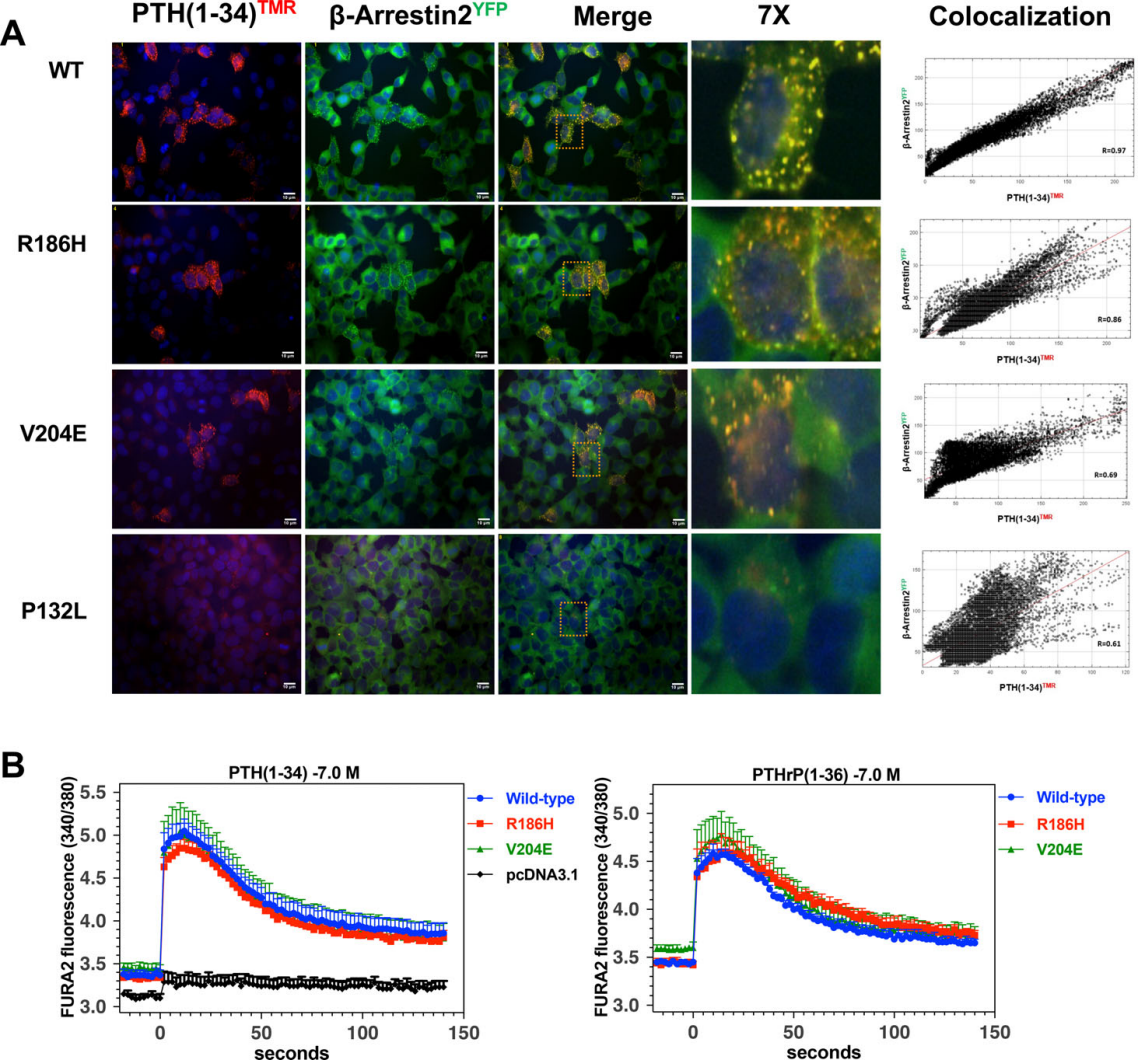
Recruitment of β‐arrestin and signaling via the Gq/PLC/IP_3_/iCa^2+^ pathway. (*A*) GBR‐24 (HEK293/GloSensor/β*‐*arrestin2^YFP^ stable) cells^(^ ^30^ ^)^ were transiently transfected to express the WT or a mutant PTH1R and then treated on coverslips with PTH(1‐34)^TMR^ (30nM) for 30 minutes at room temperature. The cells were then rinsed, fixed, stained with DAPI, and imaged using a fluorescence microscope (magnification = ×400). Areas enclosed in dashed boxes are shown digitally enlarged 7×. ImageJ‐derived colocalization plots of β‐arrestin2^YFP^ and with PTH(1‐34)^TMR^ in individual cells, with corresponding Pearson correlation coefficients (*R*), are shown in the rightmost columns. (*B*) Analysis of iCa^2+^ signaling was assessed in transiently transfected GS‐22a cells using the Ca‐sensitive fluorophore Fura2‐AM. After preloading the cells with Fura2‐AM, baseline ratiometric fluorescence (sequential excitation at 340 nm and 380; emission at 515 nm) was measured in a PerkinElmer Envision plate reader for 20 seconds prior to (baseline) and for 140 seconds after addition of PTH(1‐34) or PTHrP(1‐36) each at a concentration of 100nM. Shown are the means (±SEM) of data from five or four (PTHrP(1‐36) ligand) separate experiments.

The activated PTH1R is known to recruit beta arrestin proteins, which in turn promote receptor internalization and receptor desensitization, but may also trigger or sustain noncanonical signaling responses.^(^ ^34^, ^35^ ^)^ We evaluated β‐arrestin recruitment for the disease‐associated PTH1R‐R186H, PTH1R‐V204E, and PTH1R‐P132L, mutants using an HEK293‐derived cell line (GBR‐24)^(^ ^30^ ^)^ in which the cells stably express a YFP‐tagged β‐arrestin‐2 variant, and were further transiently transfected with the WT PTH1R or a mutant variant. Two‐days after receptor transfection, the cells were treated with PTH(1‐34)^TMR^ for 30 minutes and then, after fixing, imaged by fluorescence microscopy. In cells transfected with either PTH1R‐WT or PTH1R‐R186H and we observed a robust binding of PTH(1‐34)^TMR^ and a clustering and colocalization of the ligand with β‐arrestin2^YFP^ (Pearson correlation coefficients obtained from a representative cell were *R* = 0.97 and *R* = 0.86 for WT and R186H, respectively, Fig. 4 *A*, Supplemental Table S4). Cells transfected with PTH1R‐V204E also exhibited PTH(1‐34)^TMR^ binding as well as recruitment of β‐arrestin2^YFP^ into clusters, but colocalization (*R* = 0.69) was lower than that observed with PTH1R‐WT. Only faint signal for PTH(1‐34)^TMR^ binding and β‐arrestin2^YFP^ recruitment could be detected in cells transfected with PTH1R‐P132L (Fig. 4*A*).

The PTH1R can also couple to the Gαq/PLC/IP_3_/iCa^++^ signaling cascade, albeit generally with less efficiency than to the Gαs/adenylyl cyclase/cAMP cascade.^(^ ^36^ ^)^ We assessed intracellular Ca^++^ signaling for the PTH1R‐R186H and PTH1R‐V204E mutants, as well as PTH1R‐WT, using the calcium‐sensitive fluorophore, Fura2‐AM. GS‐22A cells transiently transfected with each receptor exhibited robust and comparable increases in Fura2‐AM fluorescence following stimulation with a single, near saturating dose (100nM) of either PTH(1‐34) or PTH(1‐36) (Fig. 4*B*).

### Responses of mutant receptors to N‐terminal PTH and PTHrP analogs

**Fig. 5:**
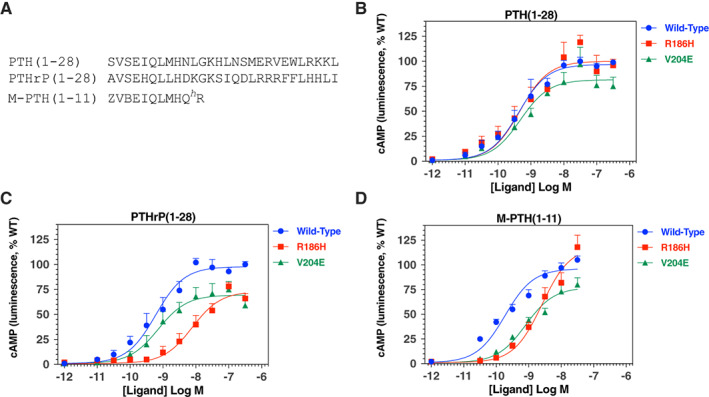
cAMP signaling responses of WT and mutant PTH receptors to N‐terminal PTH fragment analogs. (*A*) Amino acid sequences of the PTH(1‐28), PTHrP(1‐28), and M‐PTH(1‐11) analog peptides used to assess cAMP signaling responses at the WT and mutant PTH1Rs (Z = aminocyclopetylcarboxyl, B = aib, H = homoarginine). (*B*–*D*) cAMP responses to the peptide analogs were assessed in GS‐22a cells transiently transfected to express PTH1R‐WT, PTH1R‐R186H, or PTH1R‐V204E. (*B*) cAMP responses to varying concentrations of PTH(1‐28). (*C*) cAMP responses to varying concentrations of PTHrP(1‐28). (*D*) cAMP responses to varying concentrations of M‐PTH(1‐11). For each receptor and ligand concentration, the peak cAMP‐dependent luminescence response, occurring 10–20 minutes after ligand addition, was normalized to the maximum peak response observed for that ligand on PTH1R‐WT (100%) and is plotted versus ligand concentration. Data are means (±SEM) of four or three (M‐PTH(1‐11) analog) analog) experiments. Curves were fit to the data by nonlinear regression analysis; the corresponding potency and maximum luminescence values are reported in Table 2.

To further probe potential changes in receptor function, we utilized shorter‐length N‐terminal fragment peptides of PTH or PTHrP; i.e., unmodified PTH(1‐28) and PTHrP(1‐28), as well as a modified M‐PTH(1‐11) analog peptide (Fig. 5*A*). We used these shorter peptides because they presumably interact with fewer contact sites in the receptor than longer‐length peptides such as (PTH(1‐34) and PTHrP(1‐36)) and therefore would be more sensitive to perturbations in the binding site of the receptor caused by mutations, particularly in the context of transfected cells expressing the receptors at levels higher than would occur in native target cells in vivo. Moreover, the capacity of the C‐terminally truncated peptides to engage the PTH1R relies strongly, or exclusively for the M‐PTH(1‐11) analog, on interactions that occur within the transmembrane domain (TMD) bundle region of the receptor, which is where the R186H and V204E mutations occur. The fragment peptides thus differ from the PTH(1‐34) and PTHrP(1‐36) peptides that each rely on interactions to both the ECD and TMD regions of the PTH1R to bind to and maintain stability on the PTH1R.^(^ ^23^ ^)^ We surprisingly observed no difference in the cAMP responses induced by PTH(1‐28) on the PTH1R‐R186H, as compared to on PTH1R‐WT, whereas the response induced by this ligand on PTH1R‐V204E tended to be only modestly reduced in efficacy (Fig. 5 *B*, Table 2). In contrast, with the PTHrP(1‐28) peptide, the response on PTH1R‐R186H was markedly (by ~13‐fold) and significantly weaker in potency versus that on PTH1R‐WT (pEC_50_s = 8.06 ± 0.08 versus 9.16 ± 0.27, respectively, *p* = 0.016) and was reduced by ~25% for efficacy (Emax = 75% ± 4% versus 100%, respectively, *p* = 0.001), whereas the responses induced by this ligand on PTH1R‐V204E were unchanged for potency but again significantly reduced in efficacy (Emax = 72% ± 6% versus 100%, respectively, *p* = 0.003; Fig. 5 *C*, Table 2). The relative responses induced by PTH(1‐28) and PTHrP(1, 2, 3, 4, 5, 6, 7, 8, 9, 10, 11, 12, 13, 14, 15, 16, 17, 18, 19, 20, 21, 22, 23, 24, 25, 26, 27, 28) on PTH1R‐R186H therefore differed from those obtained on this mutant receptor with the intact peptides, as the Emax of the response to PTH(1‐34) was increased by ~18% and potency tended to be approximately threefold weaker, whereas the response to PTHrP(1‐36) was unchanged for efficacy and approximately twofold stronger in potency. These differences suggest that residues in the 29–34 and 29–36 regions of PTH(1‐34) and PTHrP(1‐36), respectively, can modulate the impact that the R186H mutation located in TM1 of the receptor has on the overall ligand interaction process.

**Table 2:** cAMP dose‐response analysis of N‐terminal PTH analogs on WT and mutant PTH receptors

		
	pEC_50_				Emax, %		
PTH(1‐28)			*P vs. WT*			*P vs. WT*	
Wild‐Type	9.11 ± 0.33	(0.77nM)			100 ± 0		
R186H	9.24 ± 0.11	(0.57nM)	*0.80*		98 ± 10	*0.84*	
V204E	9.35 ± 0.27	(0.44nM)	*0.61*		78 ± 10	*0.082*	

PTHrP(1‐28)			*P vs. WT*	*P vs. PTH(1‐28)*		*P vs. WT*	*P vs. PTH(1‐28)*
Wild‐Type	9.16 ± 0.27	(0.69nM)		*0.91*	100 ± 0		*0.71*
R186H	8.06 ± 0.08	(8.76nM)	*0.016*	*0.03*	75 ± 4	*0.0007*	*0.08*
V204E	9.10 ± 0.18	(0.80nM)	*0.85*	*0.50*	72 ± 6	*0.003*	*0.61*

M‐PTH(1‐11)			*P vs. WT*			*P vs. WT*	
Wild‐Type	9.72 ± 0.02	(0.19nM)			100 ± 0		
R186H	8.55 ± 0.15	(2.82nM)	*0.001*		126 ± 14	*0.14*	
V204E	9.12 ± 0.14	(0.76nM)	*0.015*		80 ± 11	*0.14*	

Cyclic‐AMP‐dependent luminescence responses to PTH(1‐28), PTHrP(1‐28) or M‐PTH(1‐11) were assessed in GS‐22a cells expressing either the WT or a mutant PTHR1. Potency values, as the negative logarithm of the half‐maximal effective ligand concentration (pEC50) with the corresponding nM concentration in parentheses, and the response maximum (Emax), as a percent of the maximum luminescence response observed for each ligand on PTHR1‐WT, were derived from curve fitting dose‐response data to a sigmoidal nonlinear regression equation. The maximum luminescence response for cells transfected with PTHR1‐WT was 135,587 ± 18,746 counts per second (cps) for PTH(1‐28), 169,037 ± 34,206 cps for PTHrP(1‐28) and 54,017 ± 4,933 cps for M‐PTH(1‐11). Values are means of four or three (M‐PTH(1‐11) analog) experiments, each performed in duplicate. The basal luminescence observed in unstimulated cells was 1,284 ± 190 cps, 2,231 ± 560 cps and 1,339 ± 173 cps for cells transfected with PTHR1‐WT; PTHR1‐R186H, and PTHR1‐V204E, respectively (P mutant vs. WT > 0.1). P values are Student T test comparisons to PTHR1‐WT or PTH(1‐28).

Testing with the M‐PTH(1‐11) analog revealed that relative to the responses on PTH1R‐WT, potency was reduced by ~15‐fold on PTH1R‐R186H and by approximately fourfold on PTH1R‐V204E (pEC_50_s = 9.72 ± 0.02; 8.55 ± 0.15, and 9.12 ± 0.14, respectively, *p* ≤ 0.015), whereas the corresponding Emax values for the responses tended to be increased on PTH1R‐R186H and decreased on PTH1R‐V204E (Fig. 5 *D*, Table 2), as found for each other ligand tested on this mutant PTH1R.

### Ligand‐binding properties of WT and mutant PTH receptors

**Fig. 6:**
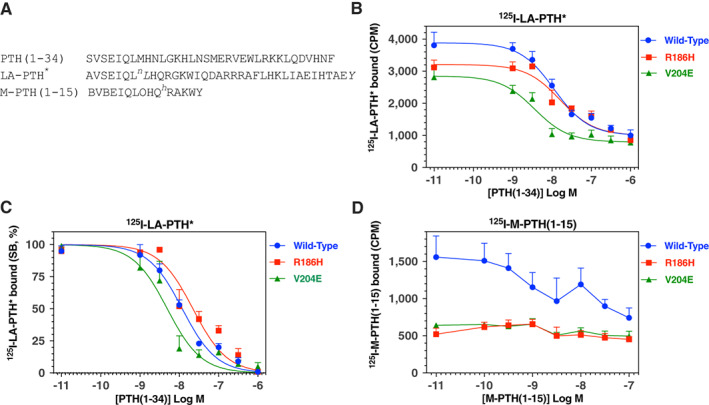
Ligand binding properties of WT and mutant PTH receptors. (*A*) Amino acid sequences of relevant peptides (^n^L = norleucine, ^h^R = homoarginine, B = Aib). (*B*) Competition radioligand binding assays performed in intact GS22a cells transfected with the WT or the indicated mutant PTH receptors using ^125^I‐LA‐PTH* as tracer radioligand and unlabeled PTH(1‐34) as competitor. The total binding of radioligand as counts per minute (CPM) is plotted versus the concentration of competing ligand. For each receptor, the total binding observed at PTH(1‐34) concentrations of 1 × 10^−8^M and higher was significantly different (*p* < 0.05) from the total binding observed in the absence of unlabeled competitor. (*C*) Data from the experiment of *B* plotted with specific binding normalized to the maximum SB observed at each receptor (100%). (*D*) Competition radioligand binding assays performed in intact GS22a cells transfected with the WT or the indicated mutant PTH receptors using ^125^I‐M‐PTH(1‐15) as a tracer radioligand and varying concentrations of M‐PTH(1‐15) as competitor. Data are plotted as in *B*. Total binding was significantly different (*p* < 0.05) from total binding observed in the absence of unlabeled competitor only on PTHR1‐WT and at the highest concentration of unlabeled M‐PTH(1‐15). Data are means (±SEM) of four experiments, each performed in duplicate. pIC_50_ and maximum binding parameters obtained from curve fitting the data obtained with ^125^I‐LA‐PTH* are reported in Table 3.

We then evaluated the R186H and V204E PTH1R mutants for the capacity to directly bind PTH peptide ligands by performing radioligand competition binding assays. Assays were performed in intact monolayers of GS‐22A cells transiently transfected with either PTH1R‐WT or a mutant PTH1R, and reactions were incubated at 4°C for 90 minutes in the presence of a tracer radioligand and varying concentrations of an unlabeled competitor ligand. We first used ^125^I‐LA‐PTH* as a tracer radioligand; this peptide is derived from a long‐acting PTH/PTHrP hybrid analog that forms highly stable complexes with the PTH1R, and similar to PTH(1‐34), interacts with both the ECD and TMD portions of the receptor^(^ ^23^, ^37^ ^)^ (Fig. 6*A*). Moreover, LA‐PTH binds with high affinity to receptors in both G protein‐coupled and G protein‐uncoupled conformational states. The use of this ^125^I‐LA‐PTH* radioligand thus permits evaluation of binding interactions occurring over the entire surface of the receptor's ligand‐binding pocket and within at least two functionally distinct receptor conformations. The maximum binding levels attained by ^125^I‐LA‐PTH* on the PTH1R‐R186H and PTH1R‐V204E mutants were reduced to about ~80% of that observed on PTH1R‐WT (*p* < 0.002, Fig. 6 *B*, Table 3); findings that are consistent with a lower binding affinity and/or a lower level of expression of the mutant receptors. Unlabeled PTH(1‐34) inhibited the binding of ^125^I‐LA‐PTH* to PTH1R‐WT with an apparent affinity of ~10nM (pIC_50_ = 7.98 ± 0.08). The apparent affinity measured in these assays for PTH(1‐34) on the PTH1R‐R186H mutant was approximately twofold weaker than that observed on PTH1R‐WT (pIC_50_s = 7.56 ± 0.06 versus 7.98 ± 0.08, *p* = 0.002), whereas the apparent affinity measured on the PTH1R‐V204E mutant tended to be approximately twofold stronger than that on PTH1R‐WT, although the difference was not significant (Fig. 6*B*,*C*, Table 3).

**Table 3:** PTH Ligand‐Binding Properties of WT and Mutant PTH Receptors

	^125^I‐LA‐PTH* versus PTH(1‐34)			
Receptor	pIC_50_ (nM)	*p* versus WT	SB (%)	*p* versus WT
WT	7.98 ± 0.08 (10.5nM)		100 ± 0	
R186H	7.56 ± 0.06 (27.3nM)	0.002	80 ± 4	0.002
V204E	8.36 ± 0.20 (4.35nM)	0.2	76 ± 4	0.001

Competition radioligand binding assays were performed in intact GS22a cells transfected with the WT or indicated mutant PTH receptor using ^125^I‐LA‐PTH* as a tracer radioligand and unlabeled PTH(1‐34) as competitor. Half‐maximal inhibitory concentrations of competing ligands, as the pIC_50_ and the corresponding nanomolar (nM) concentration in parentheses, and maximum specific binding (SB) of radioligand, as a percentage of the SB at PTH1R‐WT, were derived from curve‐fitting concentration–response curves to a sigmoidal dose–response equation. The maximum SB, as radioactive counts per minute (cpm), obtained was 2961 ± 200 cpm, 2364 ± 162 cpm, and 2269 ± 254 cpm for ^125^I‐LA‐PTH* at PTH1R‐WT, PTH1R‐R186H, and PTH1R‐V204E. The nonspecific binding (subtracted) was 831 ± 79 cpm. Data are means (±SEM) of four experiments, each performed in duplicate; *p* values are Student's *t* test comparisons to PTH1R‐WT.

To probe binding interactions occurring more specifically within the membrane‐spanning portion of the receptor, we used the radiolabeled N‐terminal PTH fragment, ^125^I‐M‐PTH(1‐15), which binds selectively, if not exclusively to the TMD region of the PTH1R and only to receptors in a G‐protein‐coupled conformation.^(^ ^37^, ^38^ ^)^ Because the R186H and V204E mutations alter residues in helix 1 of the receptor's TMD bundle, we anticipated that they would have greater impact on the binding of ^125^I‐M‐PTH(1‐15), as compared to the binding of ^125^I‐LA‐PTH*. This was indeed the case, as significant specific binding of ^125^I‐M‐PTH(1‐15) and its competitive inhibition by unlabeled M‐PTH(1‐15) was observed in GS‐22a cells expressing PTH1R‐WT, whereas no such binding was observed in cells expressing either the R186H or V204E PTH1R mutants (Fig. 6*D*).

## Discussion

**Fig. 7:**
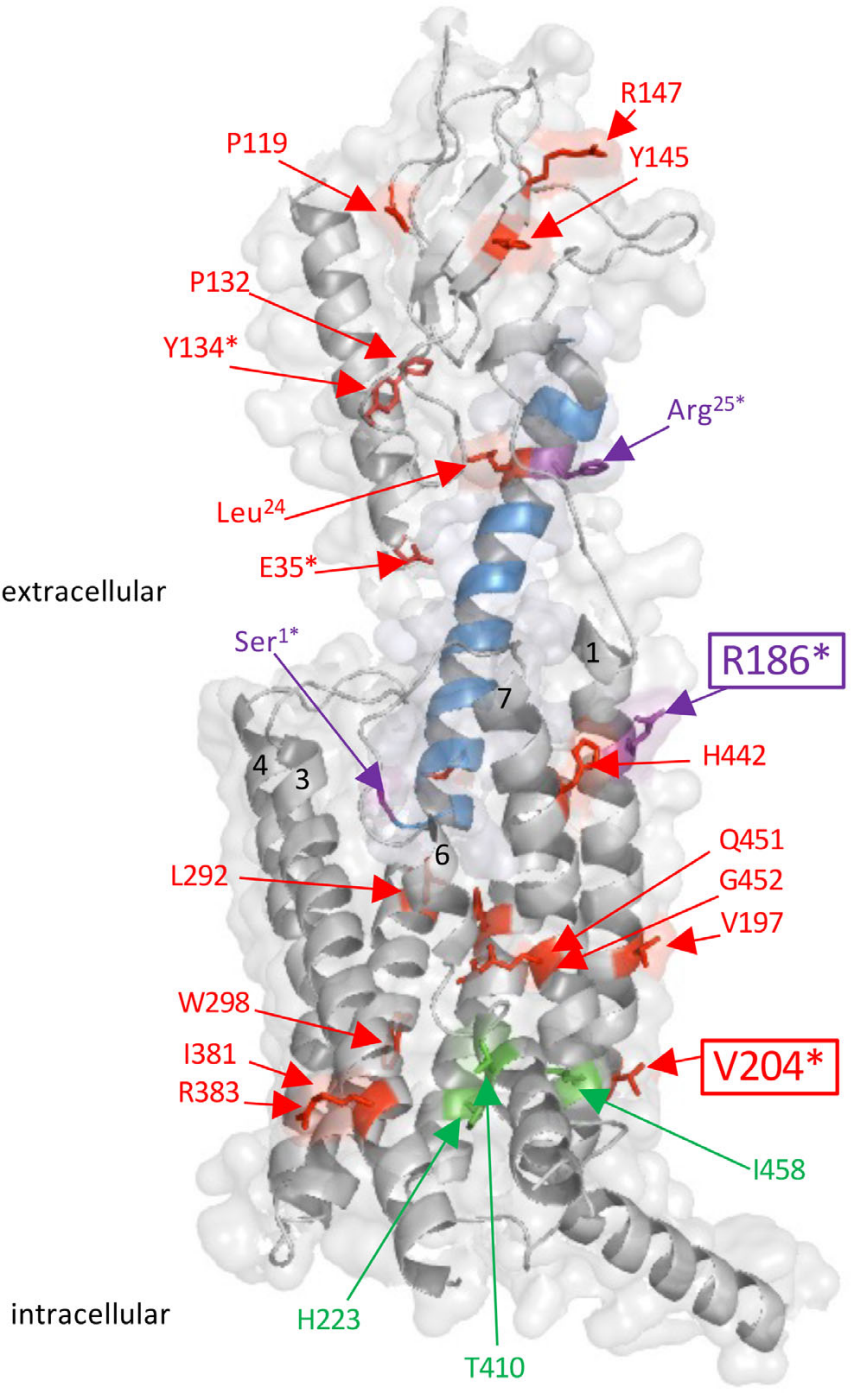
Sites of disease‐associated missense mutations in the PTH1R and PTH or PTHrP ligands. The cryogenic electron microscopy (cryo‐EM) structure (Protein Data Bank [PDB] file 6nbf) of the PTH1R in complex with LA‐PTH is shown in cartoon format with the receptor scaffold shaded gray and the ligand shaded blue. Residues at which missense mutations have been identified in patients are shown with side‐chains in stick format and colored orange if mutations are associated with dental and/or skeletal abnormalities, including a primary failure of tooth eruption (PFE): P119L, P132L, Y145C, R147C, V197E, L232R, R233H, I381S, L292P, W298V, R383Q, H442D, Q451R, and G452E^(^ ^9^, ^10^, ^11^, ^12^, ^46^ ^)^; PFE with clinodactyly: V204E^(^ ^21^ ^)^; Blomstrand osteochondrodysplasia (BOC), P132L^(^ ^4^, ^6^ ^)^; Eiken syndrome with PFE: E35K,^(^ ^18^ ^)^ Y134S,^(^ ^17^ ^)^ and brachydactyly type E with short stature and PFE: Leu8Pro and Leu24Pro in PTHrP^(^ ^47^ ^)^; residues are colored purple if the mutations are associated with alterations in calcium homeostasis resembling a pseudohypoparathyroidism (PHP) phenotype: R186H in the PTH1R, Arg25Cys^(^ ^44^ ^)^ and Ser1Pro in PTH,^(^ ^48^ ^)^ or residues are colored green if mutations are gain‐of‐function and associated with Jansen's metaphyseal chondrodysplasia: H223R, T410P/R, and I458R/K.^(^ ^49^ ^)^ Asterisks indicate residues at which mutations are homozygous (Pro132Leu is homozygous in BOC and heterozygous in PFE). Transmembrane helices 1, 3, 4, 6, and 7 are labeled at their extracellular ends by helix number. Receptor residues R186 and V204 located in TM helix 1 are sites of the homozygous mutations associated with defects in calcium homeostasis and dental/skeletal development, respectively, and studied here are boxed.

The studies presented here demonstrate that the two distinct PTH1R point mutations of R186H and V204E identified in homozygous patients with distinct phenotypes cause distinguishable changes in the functional properties of the PTH1R as assessed in vitro. These two mutations were of interest because they occurred in patients in a homozygous state, and they resulted in distinct patient phenotypes—a hypocalcemia/hyperphosphatemia phenotype similar to that seen pseudohypoparathyroidism type 1b (PHP1B) for R186H^(^ ^19^ ^)^ and a skeletal/dental phenotype for V204E in which the PFE appears similar to that seen in patients with Eiken syndrome caused by other homozygous PTH1R missense mutations.^(^ ^17^, ^18^, ^21^ ^)^ The structural model of the PTH1R‐ligand complex shown in Fig. 7 highlights various point mutations in the PTH1R and PTH and PTHrP ligands that have been identified in patients. The majority of the PTH1R mutations are heterozygous and associated with PFE, whereas relatively few are found in the homozygous state and result in the distinct pathologic conditions of BOC (P132L), Eiken syndrome (E35K and Y134S), and those of the patients with the R186H and V204E mutations studied here.

The finding that PFE occurs in both the patients with the V204E mutation and with the Eiken syndrome mutations^(^ ^17^, ^18^ ^)^ is consistent with a deficiency of the mutant PTH1R in responding to PTHrP during tooth development.^(^ ^15^, ^16^ ^)^ Tooth eruption and skeletal formation are coordinated processes that require paracrine and autocrine signaling dependent in part on local concentrations of PTHrP.^(^ ^16^, ^39^ ^)^ That the patients with the homozygous R186H mutation exhibit marked hypocalcemia without any reported dental or skeletal or abnormality led to the suggestion that this mutation causes a selective impairment in responsiveness to PTH,^(^ ^19^ ^)^ which acts to maintain calcium homeostasis. Although our current in vitro studies do not reveal clear evidence that either the R186H or the V204E mutation selectively impairs interaction with one ligand versus the other, they at least demonstrate that the two mutations do have differential impacts on the binding of structurally distinct PTH or PTHrP probe ligand analogs, and so do not rule out the possibility that such selective effects on interaction with the endogenous PTH or PTHrP peptides do occur in vivo.

In our studies, the V204E mutation present in the family affected with skeletal and dental abnormalities caused about a twofold decrease in expression of the PTH1R in transfected HEK293‐derived cells, and associated reductions in the maximal ligand‐induced cAMP signaling responses. It seems likely that the reductions in the maximum cAMP levels observed with the V204E mutant are at least in part due to a reduced surface expression level of the mutant receptor. Although the specific role of valine‐204 in folding of the PTH1R protein and its function is not known, the reduction in receptor surface expression caused by the polar V204E mutation would be expected to result in blunting of signaling responses induced by PTHrP in target cells of developing bone and dental tissues in vivo. Such effects seem to be reflected by the phenotypes of the V204E patients,^(^ ^21^ ^)^ as well as the PFE seen in patients with PTH1R haploinsufficiency resulting from other heterozygous mutations. Such blunting effects might further contribute to at least some of the skeletal and tooth manifestations seen in patients with Eiken syndrome.^(^ ^17^, ^18^ ^)^ Interestingly, Eiken syndrome is also characterized by a marked delay in ossification, which was speculated, based on mouse studies,^(^ ^40^ ^)^ to be secondary to selective impairment in the PLC/protein kinase C (PKC)/iCa^++^ signaling pathway.^(^ ^1^ ^)^ Delayed ossification was not reported for the patients with the V204E mutation, and our intracellular calcium signaling data did not reveal a defect in signaling through this pathway. Although our data do not support a major impact on the PLC/PKC/iCa^++^ signaling pathway for the V204E and R186H mutations, we cannot exclude the possibility that they can cause subtle changes in the G protein subtype coupling specificity of the PTHR1 which contribute to at least some of the clinical features of the patients.

The dental and skeletal phenotype seen in the patients with the homozygous V204E mutation are consistent with a relatively mild loss‐of‐function effect, whereas the variety of other heterozygous PTH1R mutations that are associated with PFE are likely to have a more severe impact, as indeed, at least one of them, P132L, is lethal in the homozygous state.^(^ ^4^, ^6^ ^)^ Although the overall data are consistent with a need for a certain threshold level of PTH1R expression and function in cells of developing bone and teeth,^(^ ^15^, ^16^, ^41^ ^)^ it is noteworthy that none of the patients with PFE or the V204E mutation is reported to have altered serum levels of calcium, phosphate or PTH, as is seen in the patients with the R186H mutation. Presumably, in the patients with the PFE and V204E mutations, other hormonal pathways, potentially involving 1,25(OH)2‐vitamin D, are activated and compensate for a reduced level of PTH1R function to adequately maintain calcium homeostasis.

Affected members of the family with the R186H mutation presented with hypocalcemia without skeletal abnormalities, consistent with a pseudohypoparathyroidism type 1b (PHP1B) phenotype.^(^ ^19^ ^)^ PHP1B is typically caused by mutations in the *GNAS* locus that lead to epigenetic reductions in Gsα protein expression in cells of certain imprinted tissues, including renal tubule cells involved in PTH1R‐mediated control of calcium and phosphate transport.^(^ ^20^ ^)^ Until the report of the R186H mutation,^(^ ^19^ ^)^ which we studied here, no mutation in the PTH1R has been associated with pseudohypoparathyroidism.

We found in our transfected HEK293 cells that the PTH1R‐R186H mutant, as compared to PTH1R‐WT, had comparable levels of cellular expression. The cAMP response of the PTH1R‐R186H mutant to PTH(1‐34) was, however, decreased, albeit modestly (approximately threefold), in comparison to PTH1R‐WT, such that there was a significant shift in the relative potencies of PTH(1‐34) versus PTHrP(1‐36) (Fig. 1 *D*, Table 1). The findings thus revealed a modest but selective defect in the capacity of the PTH1R‐R186H mutant to respond to PTH(1‐34), in comparison to a relatively preserved responsiveness to PTHrP(1‐36). This interpretation is consistent with the hypothesis that the R186H mutation in vivo causes a selective deficiency in responsiveness to endogenous PTH but not PTHrP.^(^ ^19^ ^)^

Somewhat surprisingly, we found that the PTH1R‐R186H mutant exhibited a more significant reduction in cAMP signaling response potency to PTHrP(1‐28) than to PTH(1‐28). The reason for this apparent change in the relative impact of the receptor mutation on the interaction with the C‐terminally truncated PTH(1‐28) and PTHrP(1‐28) fragment peptides, as compared to the relative impact seen with the intact PTH(1‐34) and PTHrP(1‐36) peptides, is not clear. The findings, nevertheless, seem consistent with the notion that interactions that occur between residues in the C‐terminal portions of the PTH(1‐34) and PTHrP(1‐36) peptides and the receptor's ECD can modulate interactions that occur within the TMD region of the receptor and involve residues in the N‐terminal portions of the ligand. Evidence for such longer‐range interactions between the ECD and TMD component of the ligand‐PTH1R complex is also suggested by recent studies on the functional impact of oxidative changes at methionine‐8 in different PTH ligand analogs, which vary depending on the presence or absence of interactions in the ECD portion of the PTH1R.^(^ ^42^ ^)^ Our current data are also consistent with the model that important and specific interactions occur between residues in the (1–13) portion of the ligand and residues in at least the vicinity of Arg186 located at the extracellular end of the receptor's first transmembrane domain helix (Fig. 7), and that receptor mutations at or near this position can modulate function in ligand‐specific fashion.^(^ ^22^, ^23^, ^43^ ^)^ Of note, PTH and PTHrP differ at several N‐terminal residue positions, including position 11, which is leucine in PTH and lysine in PTHrP, and the side‐chain if this residue is predicted to project close to the Arg186 region of the receptor^(^ ^23^ ^)^ and so could contribute to any difference in receptor interaction that occurs for the two ligands.

Although it seems plausible that the relatively modest changes in PTH ligand affinity and potency that we observed for the PTH1R‐R186H mutant in vitro could translate into the PHP1B‐like phenotype of hypocalcemia and hyperphosphatemia seen in the patients with this homozygous mutation, firmer support for this hypothesis will likely require additional in vivo or clinical evaluation. This might, for example, involve a modified Ellsworth‐Howard test in which urinary cAMP and phosphate excretion responses are measured in the patients at times after PTH infusion. Such studies in animals are precluded by the current lack of an appropriate genetic model for the patient diseases. Other limitations to our findings relate to cell‐specific differences in the levels of receptor expression, as we only studied effects of the mutations in transfected HEK293 cells, in which the PTH1R expression levels are likely to be substantially higher than would occur in cells of native PTH and PTHrP target tissue in vivo, and the higher expression levels could obscure detection of subtle changes in ligand affinity or potency that nevertheless lead to significant changes in target cell physiology in patients. Such changes might also not be revealed simply by varying DNA doses during transient transfection of the cells, and we opted to evaluate the mutants on an equal gene‐dosage basis, since we expect that to reflect the condition in patients. It is also possible that the mutations alter signaling responses through pathways in cells of different target tissues other than the cAMP and iCa^++^ pathways that we investigated in our HEK293‐derived cells. It is also worth noting that our recent studies on a homozygous Arg25→Cys mutation in PTH that is associated with severe hypocalcemia revealed only subtle changes in the functional properties of a Cys^25^‐PTH(1‐34) analog in cell‐based binding and cAMP signaling assays.^(^ ^44^, ^45^ ^)^ Thus, as for the R186H‐PTH1R mutant, the in vitro analyses were limited in their capacity to capture the phenotypic properties associated with this Cys25Arg mutation in PTH. Together, the studies highlight the challenges that can arise in the effort to explain the mechanistic basis for the effects of mutations in the PTH1R or the ligands that ultimately result in substantial disturbances in calcium and bone homeostatic systems in affected patients.

In conclusion, we present the results of functional studies with two recently described homozygous mutations in the PTH1R that were discovered in patients with very different phenotypes. Despite the remarkably different patient phenotypes, only subtle changes were found in vitro using PTH(1‐34) or PTHrP(1‐36) as probe ligands, requiring the use of shorter‐length peptides to more clearly reveal changes in receptor‐ligand interaction for these mutants compared to the PTH1R‐WT. The V204E mutation results in significantly reduced PTH1R cell‐surface expression levels leading to decreased efficacy in signaling responses induced by PTHrP or PTH ligands, supporting this as a mechanism for the tooth eruption failure and skeletal malformations seen in the patients. The R186H mutation results in near‐normal cell membrane expression levels but causes an impairment of binding interactions that occur within the receptor's transmembrane domain region, suggesting this as a novel mechanism for pseudohypoparathyroidism.

## Authors contributions

Ignacio Portales‐Castillo: Conceptualization; data curation; formal analysis; investigation; methodology; writing‐original draft; writing review and editing. Thomas Dean: Data curation; formal analysis; investigation. Ashok Khatri: Investigation; methodology; formal analysis. Harald Jüppner: Conceptualization; methodology; writing review and editing; Thomas J Gardella: Conceptualization; data curation; formal analysis; investigation; methodology; writing‐original draft; writing review and editing.

## Conflict of Interest

The authors have no conflicts of interest for the generation of this manuscript.

### PEER REVIEW

The peer review history for this article is available at https://publons.com/publon/10.1002/jbm4.10604.

## Supporting information


**Supplemental Fig. S1.** Cyclic adenosine monophosphate (cAMP) signaling responses to PTH(1‐84) and PTHrP(1‐141). cAMP signaling responses to PTH(1‐84) and PTHrP(1‐141) were assessed in GS‐22a (HEK293/GloSensor) cells transiently transfected to express PTH1R‐WT, PTH1R‐R186H or PTH1R‐V204E. Time‐dependent increases in cAMP‐dependent luminescence following addition of PTH(1‐84) or PTHrP(1‐141), were measured and the peak signal observed on each receptor at each ligand concentration, occurring ~10–20 minutes after ligand addition, was normalized to the maximum peak luminescence signal obtained with each ligand on PTH1R‐WT (100%) and plotted versus ligand concentration. Cells without ligand are represented by the −12 Log M concentration. Data are means (±SEM) of three experiments. Curves were fit to the data by nonlinear regression analysis; the corresponding potency, maximum and minimum values are reported in Supplementary Table S2.
**Supplemental Fig. S2.** Fluorescent microscopy of receptor cell surface expression and hemagglutinin (HA)‐antibody binding in unstimulated GBR‐24 cells. A) GBR‐24 (HEK293/GloSensor/β*‐*arrestin2^YFP^ stable) cells were transiently transfected to express the wild‐type or a mutant PTH1R and then treated on coverslips with AlexaFluor594‐conjugated anti‐HA.11 antibody for 60 minutes at 4°C. The cells were then rinsed, fixed, stained with DAPI and imaged using a fluorescence microscope (magnification = ×400). Transfected cells stain positively for HA.11‐Alexa544 (red) along the cell perimeter, while all cells show diffuse green staining in the cytoplasm, indicating non‐recruited β*‐*arrestin2^YFP^. The rightmost column shows 5× enlarged views of the boxed regions. Robust red fluorescence is noted for PTH1R‐WT and PTH1R‐R186H, while there is decreased fluorescence intensity for PTH1R‐V204E.
**Supplemental Table S1.** Maximum cyclic adenosine monophosphate (cAMP) luminescence responses to PTH(1‐34) and PTHrP(1‐36) on WT and mutant PTH1Rs. The Emax values from the experiments presented in Fig. 1 and Table 1 are shown expressed as the maximal counts per second (cps) value obtained for each ligand on each receptor by curve‐fitting the data to a sigmoidal dose–response equation by nonlinear regression. The basal luminescence observed in unstimulated cells was 970 ± 251 cps, 2,328 ± 639 cps and 762 ± 137 cps for cells transfected with PTH1R‐WT, PTH1R‐R186H and PTH1R‐V204E, respectively. Luminescence in cells transfected with PTH1R‐P132L and treated with 0.1nM PTH(1‐34) was 1,008 ± 299 cps. Data are means of five experiments: *p* values are Student's *t* test comparisons to PTH1R‐WT.
**Supplemental Table S2.** Cyclic adenosine monophosphate (cAMP) dose–response of PTH(1‐84) and PTHrP(1‐141) on WT and mutant PTH receptors. cAMP‐dependent luminescence responses to PTH(1‐84) and PTHrP(1‐141) were assessed in GS‐22a cells expressing either the WT or a mutant PTH1R. Potency values, as the negative logarithm of the half‐maximal effective ligand concentration (pEC_50_) with the corresponding nanomolar (nM) concentration below, and the response maximum (Emax), as a percent of the maximum luminescence response observed for each ligand on PTH1R‐WT, were derived from curve fitting dose–response data to a sigmoidal nonlinear regression equation. The maximum luminescence response for cells transfected with PTH1R‐WT was 159,225 ± 33,864 counts per second (cps) for PTH(1‐84), and 201,118 ± 30,311 cps for PTHrP(1‐141). The basal luminescence observed in unstimulated cells was 430 ± 97 cps, 823 ± 39 cps, and 420 ± 103 cps for cells transfected with PTH1R‐WT, PTH1R‐R186H, and PTH1R‐V204E, respectively. Data are means (±SEM) of three experiments: *p* values are Student's *t* test comparisons to PTH1R‐WT.
**Supplemental Table S3.** ImageJ analysis of PTH(1‐34)^TMR^‐PTH1R complexes in Gs22A cells. The fluorescent microscopy images of PTH(1‐34)^TMR^‐PTH1R complexes in Gs22A cells shown in Fig. 3 were quantified for particles (presumably internalized endosomal vesicles) using ImageJ software. Six cells positive for TMR‐PTH and hemagglutinin (HA).11‐Alexa488 antibody fluorescence in the field for each receptor were analyzed for the number of particles and the mean‐gray‐scale of the particles (Intensity). The particle count was divided by the total area of each cell analyzed (particle #/Total Area). Threshold values were adjusted on a cell‐by‐cell basis to 106 ± 3, 79 ± 10, and 86 ± 8 for cells transfected with PTHR1‐WT, PTHR1‐R186H and PTHR1‐V204E, respectively. Data are means of six cells from a representative experiment: *p* values are Student's *t* test comparisons to PTHR1‐WT.
**Supplemental Table S4.** ImageJ analysis of β*‐*arrestin2^YFP^‐PTH(1‐34)^TMR^ complexes in GBR24 cells. The fluorescent microscopy images of β*‐*arrestin2^YFP^ /PTH(1‐34)^TMR^ complexes in GBR24 cells shown in Fig. 4*A* were quantified for particles (presumably internalized endosomal vesicles) using ImageJ software. Six cells in the field positive for TMR‐PTH were analyzed for the number of particles and the mean‐gray‐scale of the particles (Intensity). The particle count was divided by the total area of each cell analyzed (particle #/Total Area). Threshold values were adjusted on a cell‐by‐cell basis to 68 ± 3.1, 70 ± 7.9, 80 ± 8 and 87 ± 4.6 for cells transfected with PTHR1‐WT, PTHR1‐R186H, PTHR1‐V204E and PTHR1‐P132L, respectively. Data are means of six cells from a representative experiment: *p* values are Student's *t* test comparisons to PTHR1‐WT.
