# Supplementary material for: Functional Properties of Two Distinct PTH1R Mutants Associated With Either Skeletal Defects or Pseudohypoparathyroidism
Source: JBMR Plus. 2022 Apr 14;6(6):e10604. doi: 10.1002/jbm4.10604 (PMC9189904; doi:10.1002/jbm4.10604)

1

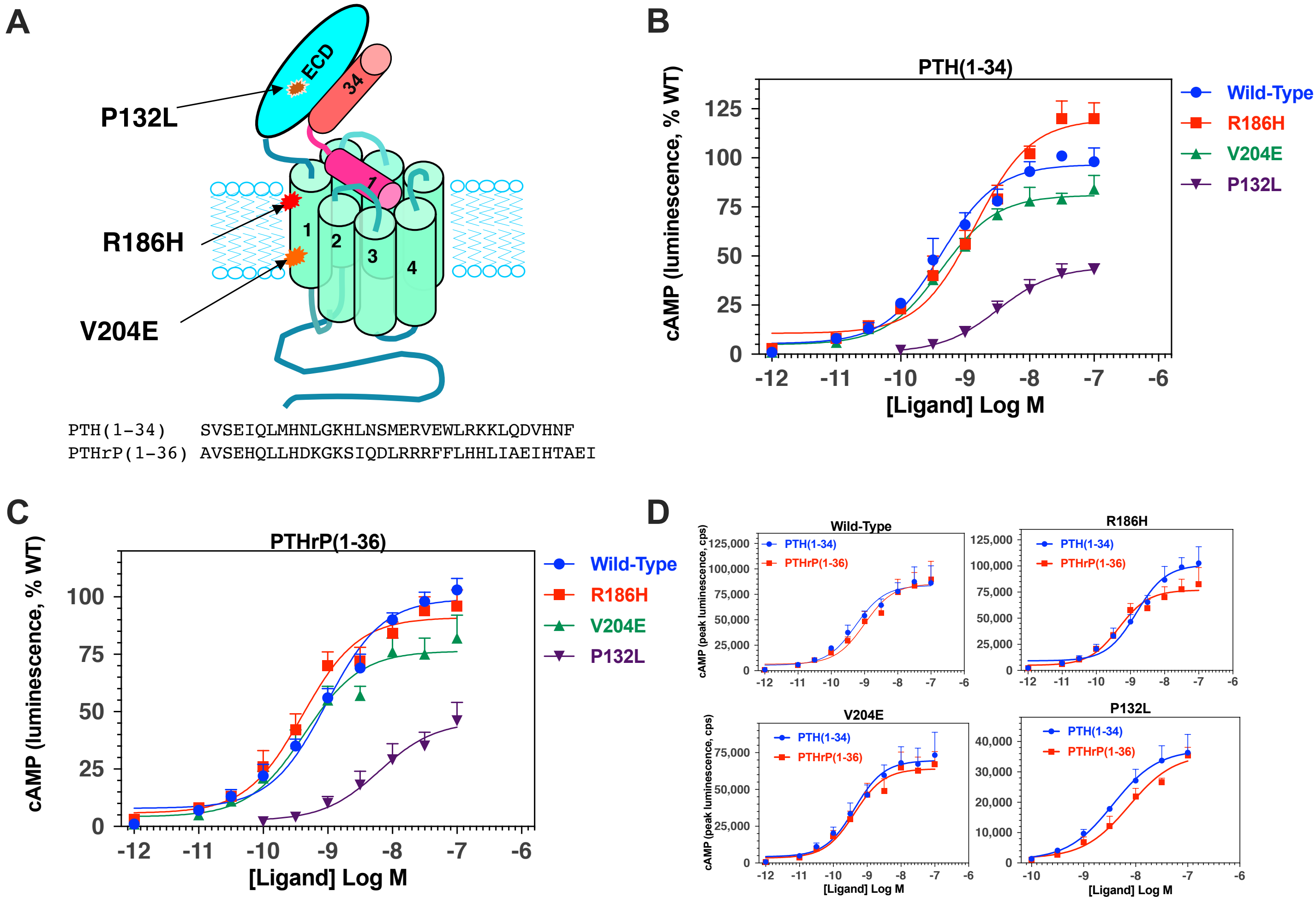
A
P132L
ECD
34
R186H
V204E
1
2
3
4
PTH(1-34) SVSEIQLMHNLGKHLNSMERVEWLRKKLQDVHNF
PTHrP(1-36) AVSEHQLLHDKGKSIQDLRRRFFLHHLIAEIHTAEI
B
PTH(1-34)
cAMP (luminescence, % WT)
[Ligand] Log M
Wild-Type
R186H
V204E
P132L
C
PTHrP(1-36)
cAMP (luminescence, % WT)
[Ligand] Log M
Wild-Type
R186H
V204E
P132L
D
Wild-Type
R186H
V204E
P132L
PTH(1-34)
PTHrP(1-36)
cAMP (peak luminescence, cps)
[Ligand] Log M

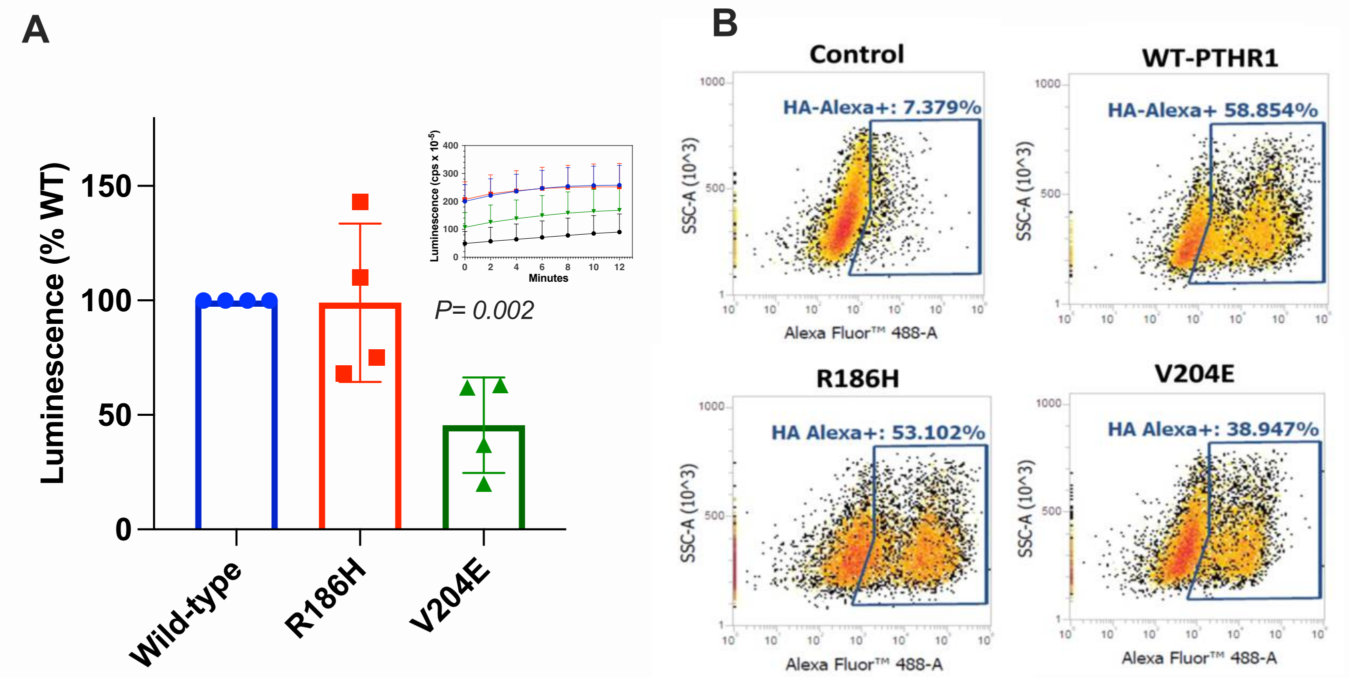

C

	Chemiluminescence anti-HA.11-HRP: % WT (cps x10^{-5})	*P vs. WT*	Flow cytometry anti-HA.11-Alexa488: Fluorescence, % WT (counts x10^{-3})	*P vs. WT*	Flow cytometry anti-HA.11-Alexa488: Gated, % WT (% total)	*P vs. WT*
**Wild-Type**	**100 ± 0**<br>*168* ± 29		**100 ± 0**<br>*25.2* ± 3.1		**100 ± 0**<br>*46.0* ± 6.1	
**R186H**	**99 ± 17**<br>*168* ± 37	*0.96*	**120 ± 7**<br>*30.2* ± 4.3	*0.057*	**107 ± 15**<br>*47.4* ± 1.5	*0.67*
**V204E**	**45 ± 11**<br>*73* ± 19	*0.002*	**28 ± 2**<br>*7.0* ± 0.6	*<0.001*	**71 ± 6**<br>*32.0* ± 2.4	*0.011*

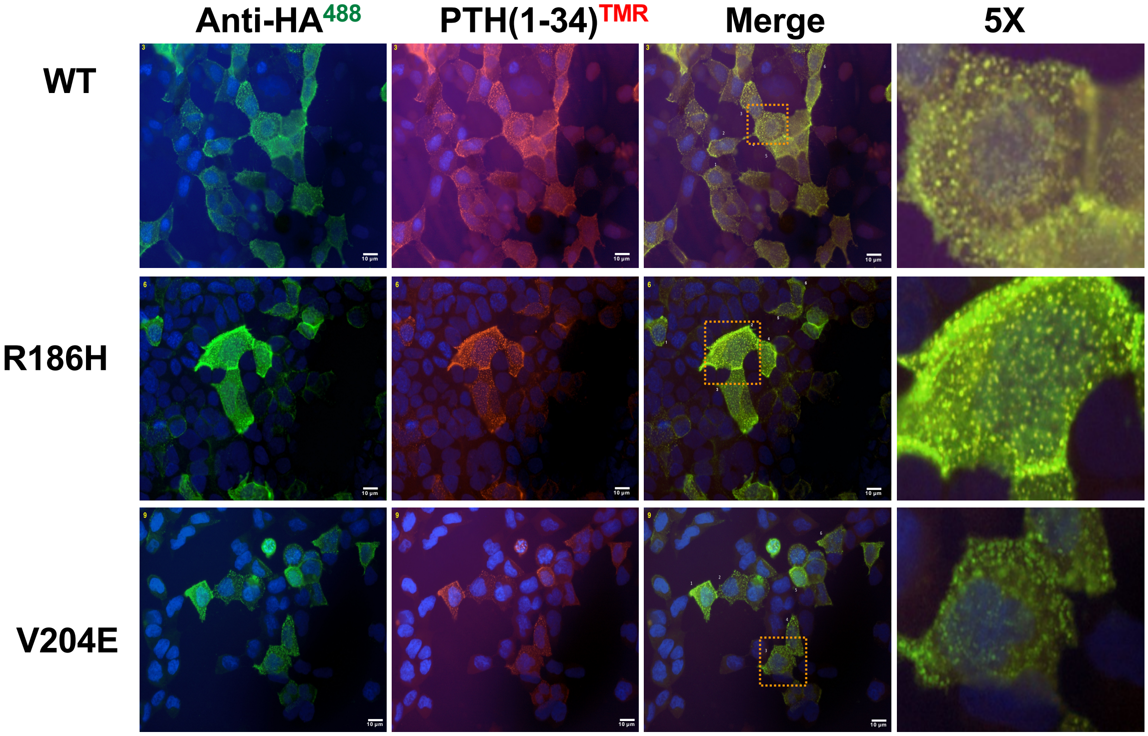
Anti-HA488
PTH(1-34)TMR
Merge
5X
WT
R186H
V204E

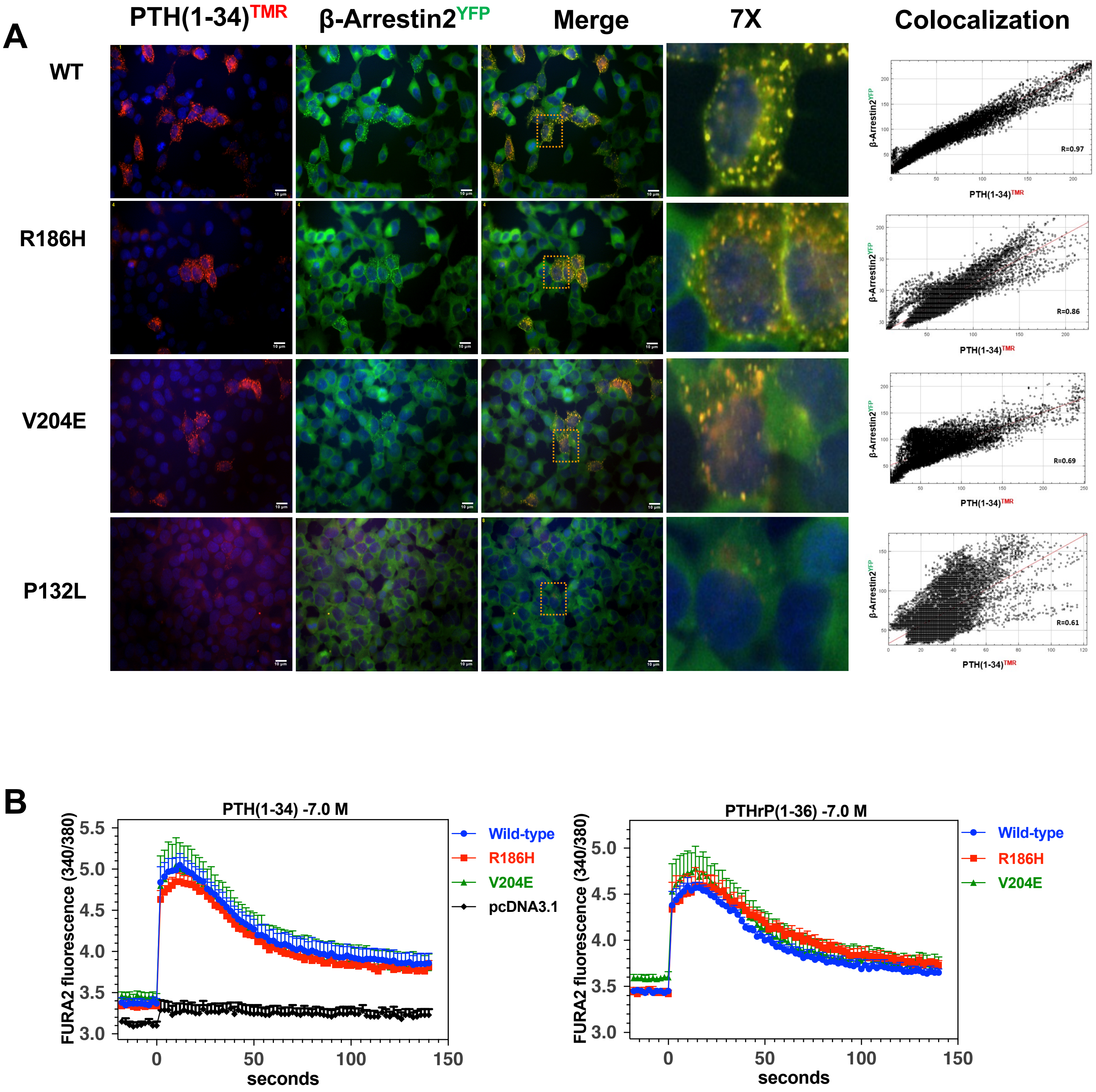
A
PTH(1-34)TMR
β-Arrestin2YFP
Merge
7X
Colocalization
WT
R186H
V204E
P132L
β-Arrestin2YFP
PTH(1-34)TMR
R=0.97
R=0.86
R=0.69
R=0.61
B
PTH(1-34) -7.0 M
FURA2 fluorescence (340/380)
seconds
Wild-type
R186H
V204E
pcDNA3.1
PTHrP(1-36) -7.0 M
FURA2 fluorescence (340/380)
seconds
Wild-type
R186H
V204E

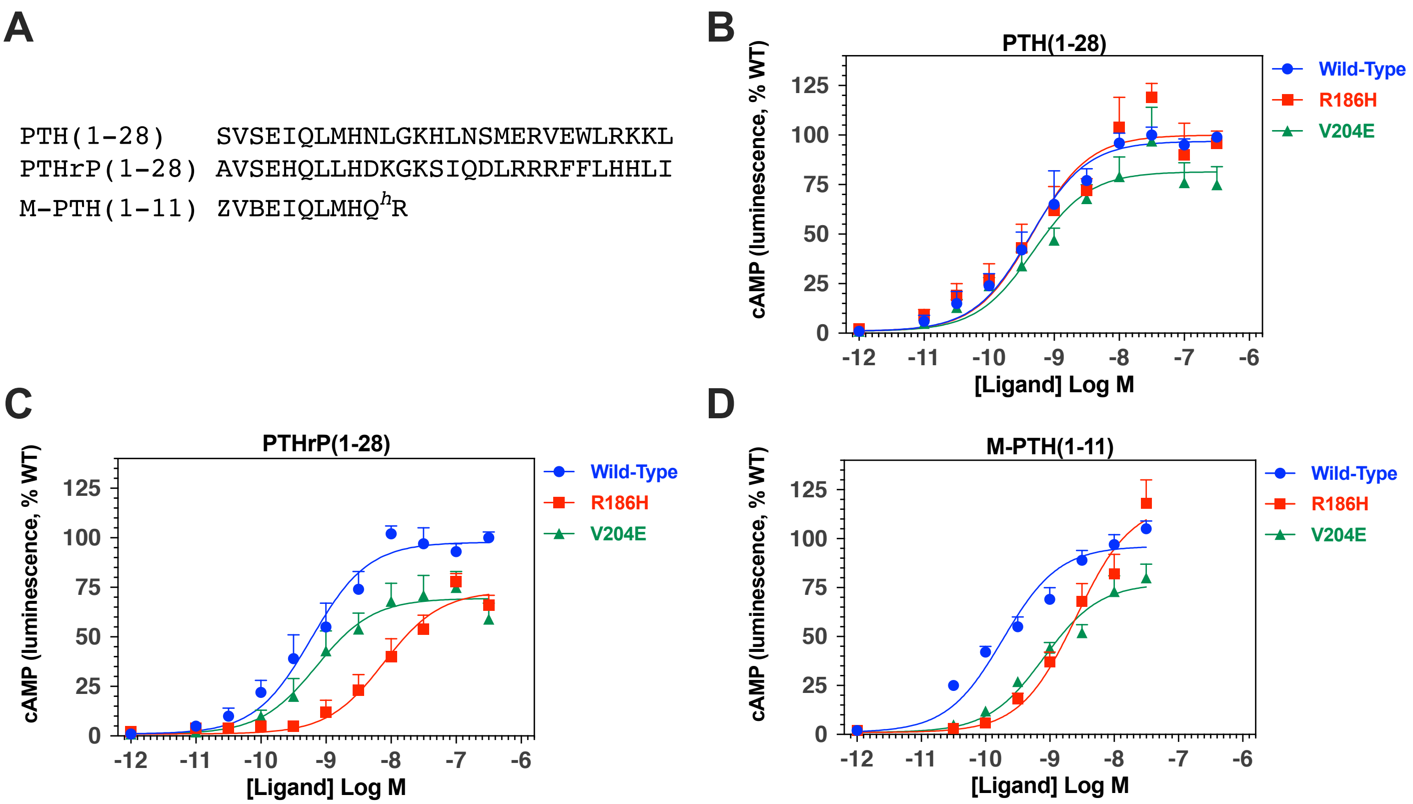
A
PTH(1-28) SVSEIQLMHNLGKHLNSMERVEWLRKKL
PTHrP(1-28) AVSEHQLLHDKGKSIQDLRRRFFLHHLI
M-PTH(1-11) ZVBEIQLMHQ^{h}R
B
PTH(1-28)
cAMP (luminescence, % WT)
[Ligand] Log M
Wild-Type
R186H
V204E
C
PTHrP(1-28)
cAMP (luminescence, % WT)
[Ligand] Log M
Wild-Type
R186H
V204E
D
M-PTH(1-11)
cAMP (luminescence, % WT)
[Ligand] Log M
Wild-Type
R186H
V204E

A

PTH(1-34)   SVSEIQLMHNLGKHLNSMERVEWLRKKLQDVHNF
LA-PTH*   AVSEIQL^n*L*HQRGKWIQDARRRAFLHKLIAEIHTAEY
M-PTH(1-15)   BVBEIQLOHQ^{h}RAKWY

B

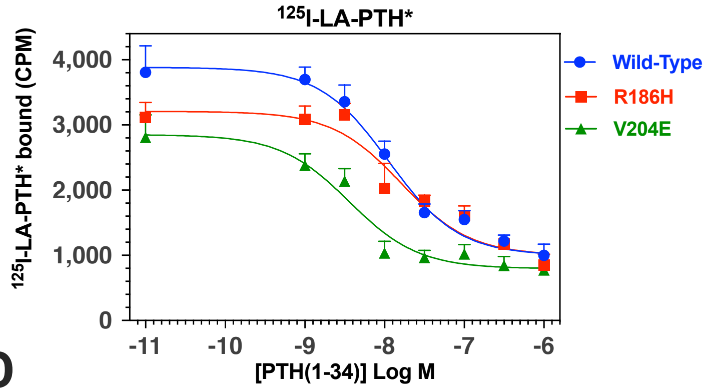

C

D

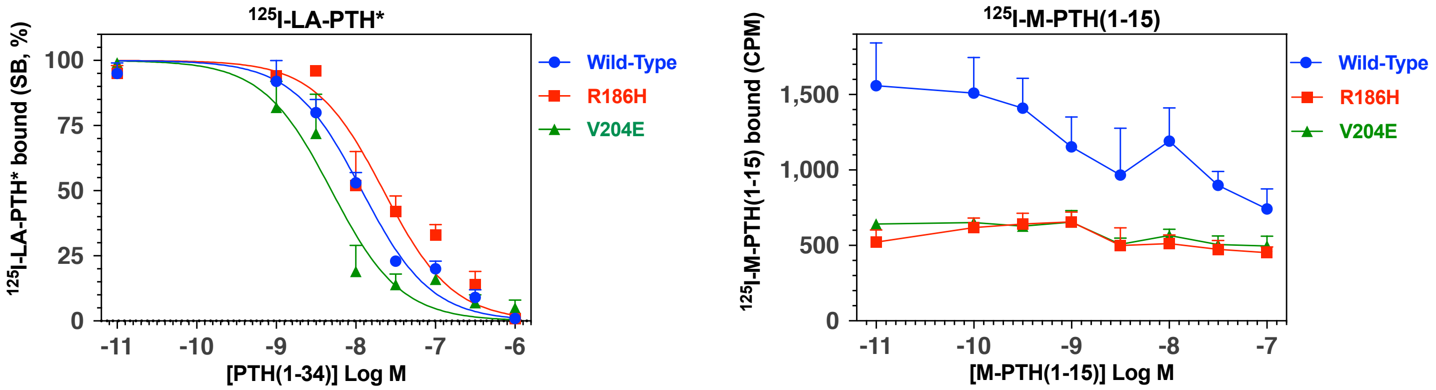

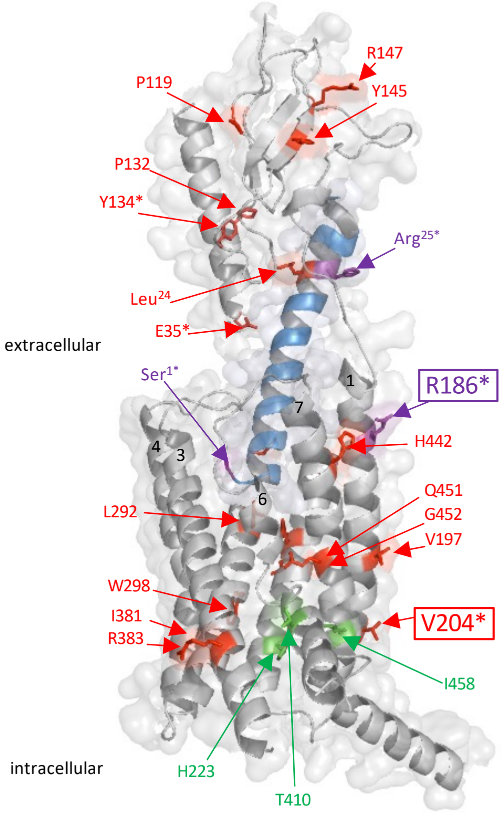
R147
P119
Y145
P132
Y134*
Arg25*
Leu24
E35*
extracellular
Ser1*
1
R186*
7
4
3
H442
6
Q451
L292
G452
V197
W298
I381
V204*
R383
I458
intracellular
H223
T410

Table 1. cAMP dose-response of PTH(1-34) and PTHrP(1-36) on WT and mutant PTH receptors

	PTH(1-34)				PTHrP(1-36)					
	pEC_{50}		$E_{max, \%}$		pEC_{50}			$E_{max, \%}$		
		P vs WT		*P vs WT*		*P vs WT*	*P vs PTH*		*P vs WT*	*P vs PTH*
**Wild-Type**	**9.36 ± 0.20**<br>*0.44 nM*		**100 ± 0**		**9.01 ± 0.09**<br>*0.98 nM*		*0.15*	**100 ± 0**		*0.69*
**R186H**	**8.92 ± 0.13**<br>*1.19 nM*	*0.11*	**118 ± 4**	*0.0024*	**9.40 ± 0.12**<br>*0.40 nM*	*0.030*	*0.028*	**96 ± 2**	*0.077*	*0.001*
**V204E**	**9.49 ± 0.13**<br>*0.33 nM*	*0.61*	**81 ± 4**	*0.0029*	**9.37 ± 0.09**<br>*0.43 nM*	*0.022*	*0.48*	**77 ± 6**	*0.0041*	*0.57*
**P132L**	**8.46 ± 0.10**<br>*3.45 nM*	*0.004*	**44 ± 4**	*<0.0001*	**8.21 ± 0.11**<br>*6.15 nM*	*0.0004*	*0.12*	**45 ± 7**	*<0.0001*	*0.95*

Table 2. cAMP dose-response analysis of N-terminal PTH fragment analogs on WT and mutant PTH receptors

	PTH(1-28)				PTHrP(1-28)						M-PTH(1-11)			
	pEC$_{50}$		Emax, %		pEC$_{50}$			Emax, %			pEC$_{50}$		Emax, %	
		P vs. WT		*P vs. WT*		*P vs. WT*	*P vs. PTH(1-28)*		*P vs. WT*	*P vs. PTH(1-28)*		*P vs. WT*		*P vs. WT*
**Wild-Type**	**9.11 ± 0.33**<br>*0.77 nM*		**100 ± 0**		**9.16 ± 0.27**<br>*0.69 nM*		*0.91*	**100 ± 0**		*0.71*	**9.72 ± 0.02**<br>*0.19 nM*		**100 ± 0**	
**R186H**	**9.24 ± 0.11**<br>*0.57 nM*	*0.80*	**98 ± 10**	*0.84*	**8.06 ± 0.08**<br>*8.76 nM*	*0.016*	*0.03*	**75 ± 4**	*0.0007*	*0.08*	**8.55 ± 0.15**<br>*2.82 nM*	*0.001*	**126 ± 14**	*0.14*
**V204E**	**9.35 ± 0.27**<br>*0.44 nM*	*0.61*	**78 ± 10**	*0.082*	**9.10 ± 0.18**<br>*0.80 nM*	*0.85*	*0.50*	**72 ± 6**	*0.003*	*0.61*	**9.12 ± 0.14**<br>*0.76 nM*	*0.015*	**80 ± 11**	*0.14*

Table 3. PTH ligand-binding properties of WT and mutant PTH receptors

	^{125}I-LA-PTH* *vs.* PTH(1-34)			
	pIC_{50} nM		SB (%)	
		P vs. WT		*P vs. WT*
**Wild-Type**	**7.98 ± 0.08**<br>*10.5 nM*		**100 ± 0**	
**R186H**	**7.56 ± 0.06**<br>*27.3 nM*	*0.002*	**80 ± 4**	*0.002*
**V204E**	**8.36 ± 0.20**<br>*4.35 nM*	*0.2*	**76 ± 4**	*0.001*

Supplemental Table 1
Maximum cAMP luminescence responses to PTH(1-34) and PTHrP(1-36) on WT and mutant PTHR1s

	PTH(1-34)		PTHrP(1-36)	
	$E_{max, cps}$		$E_{max, cps}$	
		P vs WT		*P vs WT*
**Wild-Type**	**86,291 ± 14,565**		**86,373 ± 15,167**	
**R186H**	**100,198 ± 13,491**	*0.50*	**77,242 ± 11,985**	*0.65*
**V204E**	**69,170 ± 11,137**	*0.38*	**63,481 ± 8,332**	*0.22*
**P132L**	**37,270 ± 5,993**	*0.014*	**32,347 ± 1,446**	*0.0076*

Supplemental Table 2

cAMP responses of WT and PTH1R mutants to PTH(1-84) and PTHrP(1-141) in Gs22A Cells

	PTH(1-84)				PTHrP(1-141)			
	pEC_{50}	*P*	$E_{max, \%}$	*P*	pEC_{50}	*P*	$E_{max, \%}$	*P*
**Wild-Type**	**9.05 ± 0.20**<br>*0.89 nM*		**100 ± 0**		**8.65 ± 0.10**<br>*2.24 nM*		**100 ± 0**	
**R186H**	**9.25 ± 0.11**<br>*0.56 nM*	*0.52*	**96 ± 4**	*0.49*	**8.90 ± 0.11**<br>*1.26 nM*	*0.24*	**108 ± 3**	*0.086*
**V204E**	**9.19 ± 0.12**<br>*0.65 nM*	*0.67*	**115 ± 11**	*0.37*	**8.55 ± 0.24**<br>*2.82 nM*	*0.73*	**133 ± 12**	*0.055*

Supplemental Table 3

ImageJ analysis of PTH(1-34)^{TMR}-PTH1R complexes in Gs22A cells

	Particles /Total Area	*P*	Intensity	*P*
**Wild-Type**	**1011 ± 57**		**111 ± 3**	
**R186H**	**1162 ± 95**	*0.20*	**85 ± 10**	*0.039*
**V204E**	**826 ± 78**	*0.08*	**90 ± 8**	*0.036*

Supplemental Table 4

ImageJ analysis of βArrestin^{YFP}-PTH(1-34)^{TMR} complexes in GBR24 Cells

	Particles/Total Area	*P*	Intensity	*P*
**Wild-Type**	**704 ± 22**		**81 ± 4**	
**R186H**	**603 ± 81**	*0.20*	**78 ± 9**	*0.81*
**V204E**	**496 ± 44**	*0.002*	**88 ± 8**	*0.40*
**P132L**	**223 ± 66**	*0.004*	**88 ± 5**	*0.37*

Supplemental Figure 1

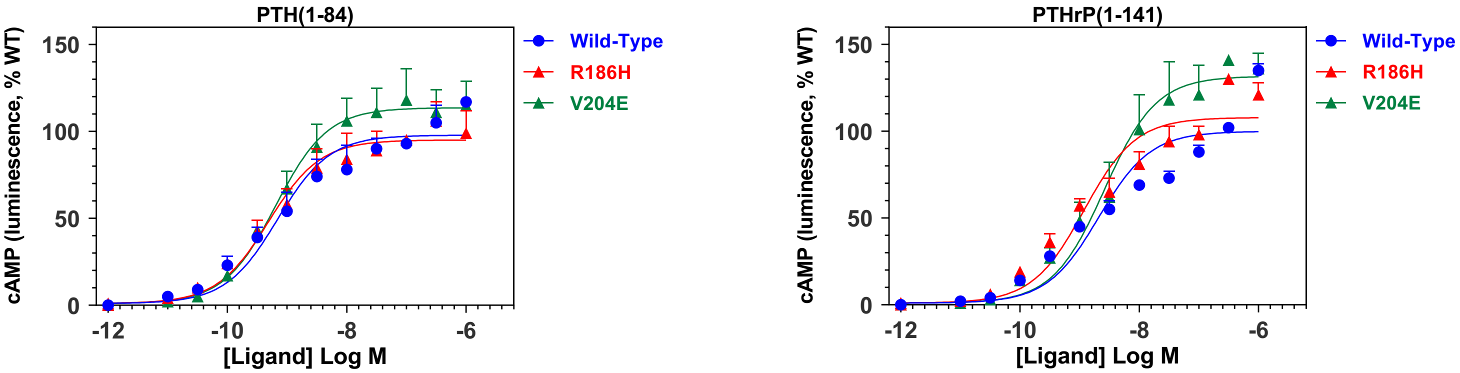

Supplemental Figure 2

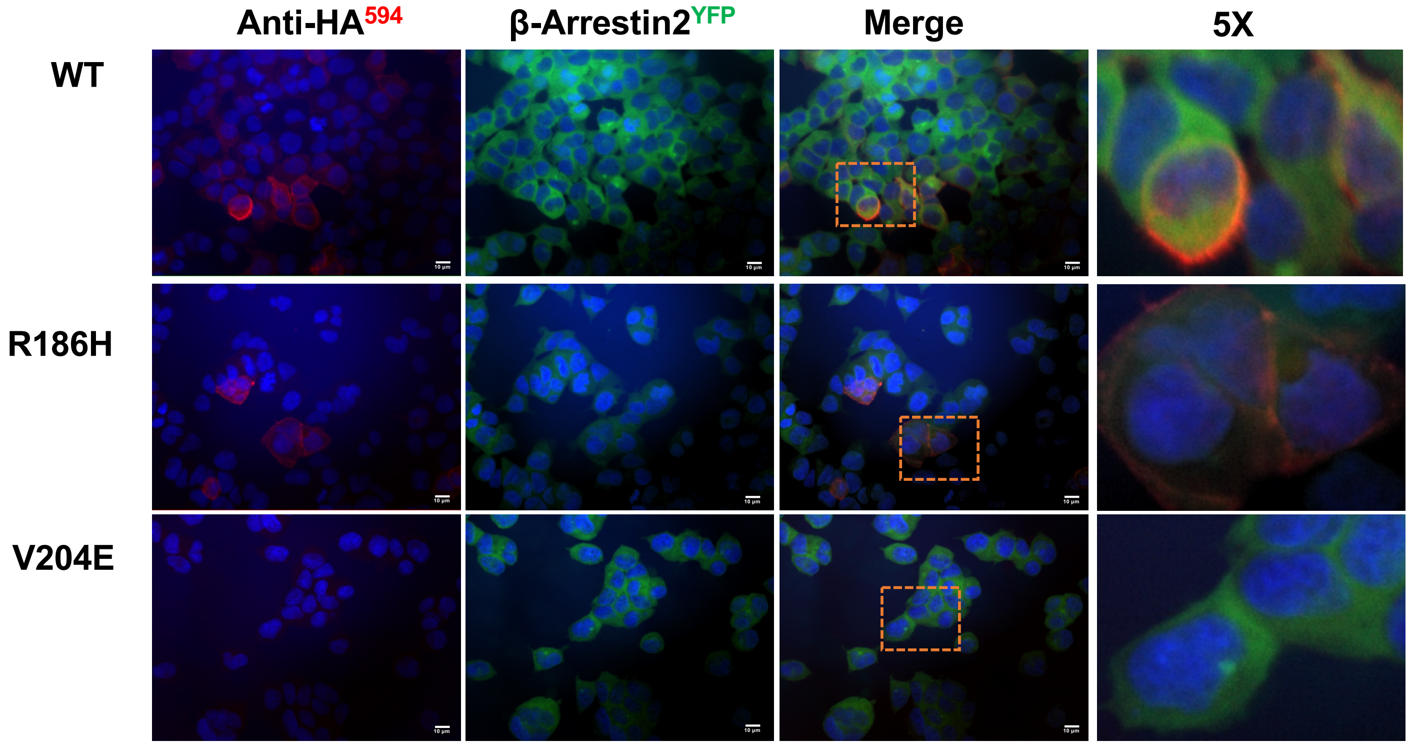

Supplement: Supplementary file 1 — Supplemental Fig. S1. Cyclic adenosine monophosphate (cAMP) signaling responses to PTH(1‐84) and PTHrP(1‐141). cAMP signaling responses to PTH(1‐84) and PTHrP(1‐141) were assessed in GS‐22a (HEK293/GloSensor) cells transiently transfected to express PTH1R‐WT, PTH1R‐R186H or PTH1R‐V204E. Time‐dependent increases in cAMP‐dependent luminescence following addition of PTH(1‐84) or PTHrP(1‐141), were measured and the peak signal observed on each receptor at each ligand concentration, occurring ~10–20 minutes after ligand addition, was normalized to the maximum peak luminescence signal obtained with each ligand on PTH1R‐WT (100%) and plotted versus ligand concentration. Cells without ligand are represented by the −12 Log M concentration. Data are means (±SEM) of three experiments. Curves were fit to the data by nonlinear regression analysis; the corresponding potency, maximum and minimum values are reported in Supplementary Table S2. Supplemental Fig. S2. Fluorescent microscopy of receptor cell surface expression and hemagglutinin (HA)‐antibody binding in unstimulated GBR‐24 cells. A) GBR‐24 (HEK293/GloSensor/β‐arrestin2YFP stable) cells were transiently transfected to express the wild‐type or a mutant PTH1R and then treated on coverslips with AlexaFluor594‐conjugated anti‐HA.11 antibody for 60 minutes at 4°C. The cells were then rinsed, fixed, stained with DAPI and imaged using a fluorescence microscope (magnification = ×400). Transfected cells stain positively for HA.11‐Alexa544 (red) along the cell perimeter, while all cells show diffuse green staining in the cytoplasm, indicating non‐recruited β‐arrestin2YFP. The rightmost column shows 5× enlarged views of the boxed regions. Robust red fluorescence is noted for PTH1R‐WT and PTH1R‐R186H, while there is decreased fluorescence intensity for PTH1R‐V204E. Supplemental Table S1. Maximum cyclic adenosine monophosphate (cAMP) luminescence responses to PTH(1‐34) and PTHrP(1‐36) on WT and mutant PTH1Rs. The Emax values from the [file JBM4-6-e10604-s001.zip › jbm410604-sup-0001-Supinfo.pdf]
